# The development and validation of a digital food-frequency questionnaire to assess habitual diet: The PREDICT Food Frequency Questionnaire

**DOI:** 10.1017/jns.2026.10080

**Published:** 2026-07-27

**Authors:** Kate M. Bermingham, Inbar Linenberg, Lucy Francis, Emily R. Leeming, Sara Megan Wallace, Fatema Badri, Alice C. Creedon, Nicky Hornzee, Hannah M. Bernard, Harry Alexander Smith, Arnab Pushilal, Joan Capdevila Pujol, Jonathan Wolf, Tim D. Spector, Wendy L. Hall, Sarah E. Berry

**Affiliations:** 1 Zoe limited, UK; 2 Department of Nutritional Sciences, King’s College London, UK; 3 Department of Twin Research and Genetic Epidemiology, King’s College London, UK

**Keywords:** Dietary assessment, FFQ, Food group intake, Nutrient intake, Validation study

## Abstract

In the context of the rapidly evolving modern food environment, there is a need for dietary assessment tools to accurately assess current food intake. The objective of this research was to develop and validate a new food frequency questionnaire (FFQ). A 265 item web-based, self-administered FFQ (PREDICT-FFQ-v1) was developed using information from two FFQs and US and UK cohort level data. Over a 1-month period, 68 participants completed the PREDICT-FFQ-v1 and six 24-h recalls (reference tool; Intake24). Relative validity was assessed using Bland–Altman plots, cross-classification food group analysis, and Spearman’s rank correlation for food groups and nutrients. From the Bland–Altman plots, the median bias between the PREDICT-FFQ-v1 and Intake24 were energy (414 kcal (95%CI 289 to 609) (1734 kj (95% CI 1208 to 2549)), carbohydrate (2.3 %EI, 95%CI 0.08 to 3.68), fat (2.5 %EI, 95%CI 0.23 to 3.52), protein (−1.14 %EI, 95% CI −1.64 to −0.23), and fibre (1.86 g/1000 kcal, 95% CI 1.05 to 2.65). Cross-classification by quartiles of diet quality (plant-based diet index) categorised >76% of participants into the same or adjacent quartiles. Correlation coefficients for food groups assessed using both tools ranged from 0.38 (legumes) to 0.78 (meat), and for energy-adjusted nutrients ranged from 0.28 (total fat) to 0.82 (alcohol). The PREDICT-FFQ-v1 captures intakes of foods that are commonly consumed in modern diets, with greater resolution at the food level than previous tools. The PREDICT-FFQ-v1 has acceptable relative validity in comparison to 24-h dietary recall and provides a valuable alternative for assessing dietary intake in nutritional epidemiology.

## Introduction

Accurate assessment of dietary intake is a complex, yet critical pillar of the relationship between dietary quality and health^(1)^. Numerous tools exist to assess dietary intake that differ in reliability and user burden for both researchers and respondents^(2)^. Objective tools for dietary assessment include the doubly labelled water method and assessment of urinary biomarkers. Both methods show strong correlations with subjects’ dietary intake and are not impacted by reporting bias; however, these methods are expensive and involve complex technical analysis that make them difficult to apply to large samples or over a longer duration^(3–5)^. Conversely, self-reported dietary assessment tools offer a lower burden option to dietary assessment, requiring participants to report their dietary intake in response to prompts using questionnaires, researcher-led interviews or food diaries, but are prone to reporting bias^(6)^. Despite this, subjective dietary assessment tools are relatively easy to implement in larger, longitudinal studies and are therefore widely used.

Food frequency questionnaires (FFQs) allow for assessment of habitual dietary intake, ranging from one month to one year^(7–10)^. FFQs can be used to assess dietary intake at both the nutrient and food levels. This facilitates a food-first approach to dietary analysis, making it possible to assess the relationship between diet and health beyond nutrient composition alone. However, the capacity of existing FFQ tools to measure food and nutrient exposure in the current varied food environment, including high resolution at the food level, is restricted. In most FFQs, food items are grouped into composite categories (e.g. whole grains, legumes and pulses, dark leafy greens, spreads). This prohibits the quantification of individual item intake and more granular assessment of associations between foods and health outcomes. This is a significant limitation given current advancements in our understanding of the chemical and structural complexity of single foods and the subsequent impact of the food matrix on nutrient bioavailability and absorption.

Given the limitations of current FFQs there is a need for a tool that captures high resolution data at the food level, allowing assessment of the relationships between foods and food groups consumed as part of modern diets and health outcomes. The primary objectives of this work were 1) to develop a self-administered, quantitative, online FFQ that captures intake of discrete dietary items commonly consumed in modern diets, with greater resolution at the food level in comparison to existing methods and 2) to determine the relative validity of the FFQ for the quantification of food and nutrient intake among adults in the UK. An average of multiple administrations of dietary recalls is considered the ‘gold standard’ in assessing habitual intake^(11)^. Therefore, the FFQ developed for this study is compared to multiple administrations of an online, self-administered 24-h recall tool Intake24 (https://intake24.co.uk) in an adult population in the UK.

## Methods

### Development of the FFQ

FFQs are commonly developed by modifying existing questionnaires, or curating new surveys from basic principles, such that the resulting tool is best suited to its application^(12)^. The PREDICT FFQ (version 1; PREDICT-FFQ-v1) was adapted from the European Investigation into Cancer and Nutrition (EPIC)-Norfolk Study FFQ^(10)^, and the Diet History Questionnaire (DHQ III), a FFQ developed by the US National Cancer Institute^(9)^. The EPIC FFQ was previously validated against intakes of energy, protein, and macronutrients assessed using the doubly labelled water method, urinary nitrogen, and repeated 24-h dietary recall methods respectively^(10)^, whilst the performance of the DHQ questionnaires in estimating nutrient intakes was previously validated against telephone-administered 24-h dietary recalls and the Block and Willett FFQs^(9)^.

To develop the new PREDICT-FFQ-v1, we identified food items commonly consumed in current UK and US diets that were not included in previous FFQs, using data from the PREDICT study cohorts; in which participants completed the EPIC FFQ (in the UK cohort), DHQ III (in the US cohort) and estimated food records (in UK and US cohorts)^(13–15)^. This allowed the identification of a list of commonly consumed foods that were not present in earlier FFQs. In addition, this allowed the inclusion of food descriptions as both US and UK food name equivalents. All foods were carefully selected by a registered nutritionist and dietitian and reviewed for their level of detail (described as a singular food, or within a food group), with a focus on including more questions about healthful foods, such as fruits, vegetables, and fermented foods, as well as those that are less commonly consumed. The researchers aimed to allow more granular analysis of individual foods compared to traditional FFQs, such as the EPIC FFQ, which has limited detail on certain items (e.g. only 36 out of 130 foods are fruits and vegetables in the EPIC FFQ). In total, 265 food and beverage items were included in the PREDICT-FFQ-v1, in comparison to the EPIC FFQ (*n* = 130 items)^(10)^ and the DHQ III (*n* = 135 items)^(9)^. Food items were included as discrete entities except where their frequency of consumption or similarity to other foods indicated that they should be grouped into composite categories, for example, fruit pies and crumbles. The additional food items included in the PREDICT-FFQ-v1, compared to the EPIC FFQ, are reported in Supplementary Table 1.

The PREDICT-FFQ-v1 was uploaded online to a survey platform (https://www.typeform.com/) which participants accessed via a link provided by study coordinators. Participants were asked which foods they had consumed in the past month only. There were both images and text descriptions of the amount of food to guide them. Participants were asked how often they ate a particular amount of food shown in an image with a text description. The participant selected how often they consumed the set amount during the assessment period (past month). If a participant did not select an item, its intake was coded as zero.

### FFQ Consumption Frequency and Portion Sizes in the PREDICT-FFQ-v1

Frequencies of food and beverage item consumption were based on levels used in the DHQ III, which included eight frequency levels for food items and nine frequency levels for beverages. Frequencies ranged from ‘once in the past month’ to ‘6 or more times per day’. Respondents were asked to report the frequency with which they consumed each food and beverage item over the preceding month. Portion sizes were applied to each item included in the FFQ and were derived from standard portions set by the Food Standards Agency (FSA)^(16)^. For example, once a month is 1/month, and 2–3 times in the past month is 2.5 times/month (see Supplementary Table 2 for all frequency levels). Portion sizes for food and beverage items that were not included in the FSA guidance were derived from the US Department of Agriculture (USDA) database^(17)^. A registered nutritionist and dietitian also provided expert opinion for portion sizes where the database was not up to date with current dietary content and habits, for example, relating to more novel products such as falafel. The registered nutritionist and dietitian also reviewed all portion sizes and adjusted based on expert opinion any portion sizes which were visually unrealistic compared to their portion size photos (e.g. differences in portion sizes of burgers as stated in USDA/FSA guidance compared to the photograph being used for the item).

Portion size options displayed to respondents were described as the functional portion (e.g. one medium apple) or in grams. Questionnaire items were accompanied by photographic images of the item (similar to that of a photographic food atlas^(18)^; displayed as full standard portion size on white crockery, alongside cutlery) or a close equivalent of the item. Every food item had a photographic image displayed to aid food quantification (see Supplementary Figure 1 for a selection of food images).

### Nutrient Composition

Nutrient intakes were calculated by multiplying the frequency of food intake by standard portion weights to obtain grams of each food consumed per day. These were then converted to nutrient intake using an appropriate food code. Food codes were identified by mapping PREDICT-FFQ-v1 foods to an appropriate food or beverage item in the McCance and Widdowson database^(19)^. Nutrient information for food and beverage items that were not included in the McCance and Widdowson database were derived from the USDA’s Food and Nutrient Database for Dietary Studies (FNDDS)^(20)^. Some FFQ items were allocated multiple constituent foods, with nutrient information given as a weighted ratio, based on average national consumption levels^(21)^. For example, the nutritional output for the question ‘How often did you have this amount of butter?’ was calculated from ratios of the foods ‘Butter, salted’, and ‘Butter, unsalted’. For foods where full-fat and low-fat options are not listed separately, they are combined into a single question (e.g. ‘How often did you use this amount of salad dressing (regular or low fat)?). The nutritional data for these items is an average of both full-fat and low-fat types.

### 24-Hour Recall

The PREDICT-FFQ-v1 was compared against an average of six administrations of a 24-h dietary recall (Intake24). The recalls were completed over two reporting periods; each consisting of three consecutive days, as multiple administrations of 24-h dietary recalls has been shown to increase their accuracy, in comparison to a single administration^(22,23)^. Intake24^(24)^ is an open source dietary assessment research tool, freely available to researchers, that is maintained and developed by the Nutrition Measurement Platform, MRC Epidemiology Unit, University of Cambridge, in collaboration with Open Lab, Newcastle University. Development of this tool has been described in detail elsewhere^(25)^. The tool prompts respondents to list all food and beverage items consumed during the previous day (from midnight to midnight) using free text entry. Entered foods and beverages are then matched to equivalent items using food composition codes in the Intake24 database, the UK Nutrient Databank. Portion size is reported through selection of a single portion size from a range of options accompanied by food photographs. Respondents are asked to review their entered items and given the option to enter any further intake before submitting their recall. Intake24 is widely used and has been validated previously against objective dietary assessment methods including validation of energy intakes against concurrent measurement of total energy expenditure using doubly labelled water^(26)^.

### Plant-Based Diet Index

The plant-based diet index (PDI)^(27)^ was calculated from both Intake24 and the PREDICT-FFQ-v1 to assess diet quality. The PDI can be computed as three separate metrics: an overall PDI, representing diet quality from consumption of all plant food while reducing animal food intake, a healthful plant-based diet index (hPDI) that represents diet quality based on intakes of healthy plant foods and an unhealthful plant-based diet index (uPDI) that represents diet quality based on less healthy plant foods known to be associated with a higher risk of several diseases^(27)^. National Diet and Nutrition Survey (NDNS) sub-food groups (*n* = 154 codes) were available for the Intake24 and the PREDICT-FFQ-v1. These food groups were mapped into 18 PDI groups to calculate the PDIs.

### Participants

Participants were recruited from the ZOE nutrition product waitlist (a cohort of UK-based adults that had registered their interest in purchasing the ZOE product (https://zoe.com/)) via email. The study recruitment target was 100, informed by previous evidence^(12,28)^ and sample size required for accurate statistical estimation^(29)^. Interested volunteers were screened for eligibility against the study inclusion criteria: 1) able to provide consent to participate; 2) not currently enrolled in another research study; 3) able to maintain their habitual diet until completing the study; and 4) based in the UK. This study was conducted according to the guidelines laid down in the Declaration of Helsinki and all procedures involving human subjects/patients were approved by the King’s College London Research Ethics Committee (Approval no. LRS/DP-22/23-33973). Electronic informed consent was obtained from all subjects. The trial was registered on ClinicalTrials.gov (NCT05612997).

### Study Design

**Figure 1.**
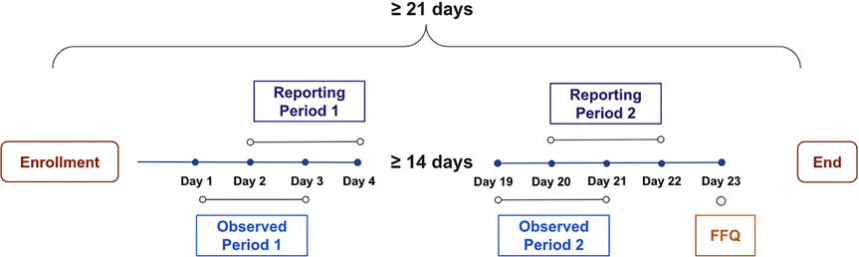
Figure 1 long description. Study design. The PREDICT FFQ (v1; PREDICT-FFQ-v1) was validated against six administrations of a 24-h dietary recall (Intake24). Intake24 measures dietary intake by asking respondents to list all foods and beverages consumed on the previous day (midnight to midnight). Dietary recalls were conducted across two reporting periods, separated by a 2-week washout. Reporting periods consisted of 3 consecutive days each; reporting period 1 took place on days 2–4, to assess dietary intake on days 1–3 (the observed period); reporting period 2 took place on days 20–22, to assess dietary intake on days 19–21. Each reporting period consisted of two weekdays and one weekend day. The FFQ was administered on day 23, to assess dietary intake over the past month.

This single-arm study consisted of a three to four week dietary assessment period, in which both assessment methods (PREDICT-FFQ-v1 and Intake24) were completed remotely (Figure 1). Participants were asked to maintain their habitual diet from the point of enrolment until the end of the study. The dietary assessment period was divided into two reporting periods, separated by a washout of at least two weeks. In the first reporting period, participants completed three 24-h recalls on days 2–4 to assess dietary intake on days 1–3. During the second reporting period participants completed a further three 24-h recalls on days 20–22 (assessing dietary intake on days 19–21) or on days 23–25 (assessing dietary intake on days 22–24). During each reporting period, 24-h recalls were completed across three consecutive days, including two weekdays and one weekend day (i.e. Sunday–Tuesday or Thursday–Saturday), in order to capture inter-day variation and enhance accuracy^(2)^.

**Figure 2.**
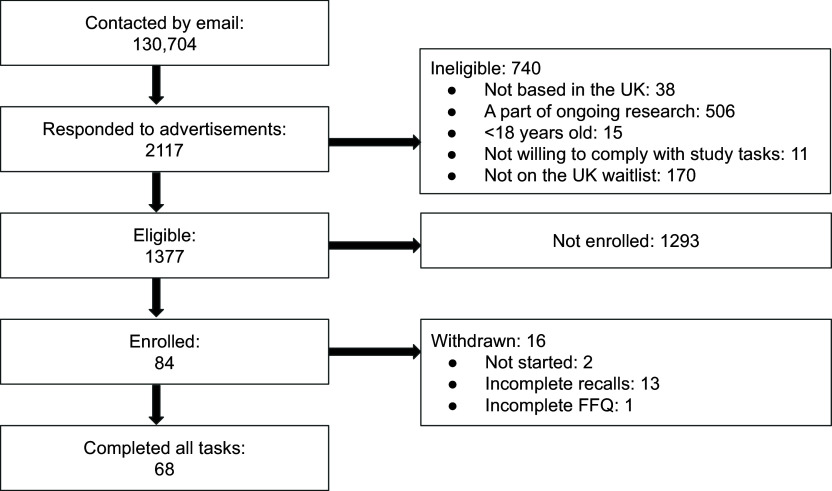
CONSORT diagram. CONSORT, Consolidated Standards of Reporting Trials.

On the day following the last Intake24 recall completion, participants completed the PREDICT-FFQ-v1 to report their dietary intake over the preceding month (encompassing the same period of intake captured by the six 24-h recalls). Completion of the PREDICT-FFQ-v1 was accepted within 14 days of the final 24-h recall. Reminders to complete study tasks on specified days were sent to participants via email. Adequate completion (before midnight on the day of recall) and resolution of reporting issues were confirmed by study staff before the next 24-h recall was administered. Participants who did not complete one or more 24-h recalls or the PREDICT-FFQ-v1, within the specified period were withdrawn (*n* = 14; Figure 2).

### Acceptability

As part of the completion of the PREDICT-FFQ-v1, participants were asked questions relating to their experience of completing the questionnaire. These included ‘How long did it take you to complete this questionnaire?’ with response options of ‘Less than 10 minutes’, ‘10-20 minutes’, ‘20-30 minutes’, and ‘More than 30 minutes’; ‘Did the images help you to estimate how much of a food you’d had?’ with responses ranging from 1 (not helpful) to 5 (very helpful); ‘What did you find clear in the questionnaire?’ with response options of ‘questions’, ‘food portion images’, ‘text description of food portion sizes’, and ‘choice of foods’; and ‘What did you find unclear in the questionnaire?’ with response options of ‘questions’, ‘food portion images’, ‘text description of food portion sizes’, ‘choice of foods’, and ‘everything was clear’.

### Statistical Analysis

All analyses were performed using Python 3.8.3, Pandas 1.5.3, SciPy 1.6.3, and R version 4.0.2 (2020-06-22). Figures were created on Graphpad Prism Version 10.3.1. Daily nutrient intakes calculated from the PREDICT-FFQ-v1 and Intake24 (average of six administrations) are presented as median, 25th and 75th percentiles. Data were visually inspected for outliers and trends. Outliers in energy intake were defined, in datasets from both tools, as those meeting both of the following criteria as per the method used in the EPIC FFQ^(10)^: 1) falling outside of 1.5 times the interquartile range of the ratio of energy intake to BMR (EI:BMR) and 2) falling inside the top or bottom 0.5% of the EI:BMR ratio. BMR was calculated using Henry equations^(30)^.

The agreement between the PREDICT-FFQ-v1 and 24-h recalls was assessed using Bland–Altman plots, to visualise the difference in nutrient intakes and food groups between the two methods plotted against the mean intake of the two methods. A nonparametric approach to comparing methods was applied^(31)^. Limits of agreement (LOAs) were calculated as the 2.5 and 97.5 percentiles, providing an interval that includes the difference between single measurements on the same participant by the two methods with 95% probability.

The Wilcoxon signed-rank test was applied to assess the differences between the estimated nutrient and food group intake from the PREDICT-FFQ-v1 and 24-h recalls. Cross-classification tables were created to evaluate the extent to which the PREDICT-FFQ-v1 classified participants into the same quartiles of dietary quality (PDI) as the Intake24 recalls. Agreement between methods is presented as the proportion of participants classified into the same/adjacent or opposite quartile by the PREDICT-FFQ-v1 and Intake24 recalls. Spearman’s rank-order correlation coefficients, reflecting the degree to which the PREDICT-FFQ-v1 and repeated 24-h recalls ranked participants equally in terms of food group and nutrient intake were assessed. In accordance with previous validation studies^(32,33)^ the following Spearman’s rho cut-offs were applied to categorise the strength of the correlations: poor <0⋅20, acceptable 0⋅20–0⋅49 and strong ≥0⋅50. Two-tailed *p*-values are presented. For all analyses, *p*-values of <0.05 were considered statistically significant. To assess the proportion of participants who may follow a vegetarian-style dietary pattern all meat, poultry, and seafood based questions were identified within the PREDICT-FFQ-v1 (Supplementary Table 3). The proportion of participants who did not consume any of these foods were classified as participants who may follow a vegetarian-style dietary pattern and are shown in Table 1.

**Table 1.** Characteristics of study participants Table 1 long description.

	Total cohort (*N* = 68)		Males		Females	
Sex (*n*, %)			17	25	51	75
Age (years)	48.3	11.2	48.7	11.7	48.2	11.2
Height (cm)	170.1	9.0	178.6	8.6	167.3	7.3
Weight (kg)	74.6	17.7	82.7	13.7	71.9	18.3
BMI (kg/m^2^)	25.7	5.6	25.8	2.8	25.7	6.3
Physical activity (min/week)	179.5	245.2	227.7	388.9	163.5	176.5
Smoking status (n, %)						
Never	38	55.9	9	52.9	29	56.9
Used To	29	42.6	8	47.1	21	41.2
Yes	1	1.4	0	0	1	1.9
Ethnicity (*n*, %)						
White	62	91.2	16	94.1	46	90.2
Asian or Asian British	5	7.4	–	–	5	9.8
Other	1	1.4	1	5.9	–	–
Education level (*n*, %)						
University degree	56	82.4	15	88.2	41	80.4
Non-university degree	12	17.6	2	11.7	10	19.6
Vegetarian-style dietary pattern (n, %)	6	9	2	12	4	8

Data are mean and standard deviation unless otherwise stated.

### Accessing the *PREDICT-FFQ-v1*

The PREDICT-FFQ-v1 questions are provided as an appendix and an online template is freely available for researchers upon request from data.papers@joinzoe.com. The scripts for the statistical analysis are freely available upon request to ZOE Ltd. Application is via data.papers@joinzoe.com. Code will be made available within 2 months of the request.

## Results

### Baseline characteristics of the study participants

Of 2117 participants assessed for eligibility, 84 were enrolled in the study and 68 successfully completed (CONSORT diagram, Figure 2). Participant characteristics are shown in Table 1. Of 68 participants, 75.0% were female. Participants had a mean (± SD) age of 48.3 years (± 11.2) and mean BMI of 25.7 kg/m^2^ (± 5.6). The majority of participants were White (91.2%) and had obtained a higher education degree (82.4%), and 1.4% reported they were smokers. There were no participants excluded based on outliers in energy intake.

### Acceptability

Upon completion of the PREDICT-FFQ-v1 questionnaire, participants were asked a number of questions relating to their experience completing the PREDICT-FFQ-v1. In response to the question ‘How long did it take you to complete the questionnaire?’ a total of 8.8% (*n* = 6) of participants reported that it took less than 10 min to complete the PREDICT-FFQ-v1, 64.7% (*n* = 44) reported between 10 and 20 min, 23.5% (*n* = 16) reported between 20 and 30 min and 2.9% (*n* = 2) reported that it took longer than 30 min to complete the PREDICT-FFQ-v1. The survey platform, typeform, also provided the average time (22.1± 23.3 min) taken to complete the PREDICT-FFQ-v1. Participants were asked how helpful the images were in estimating food intake, using the question ‘Did the images help you to estimate how much of a food you’d had?’ and answered on a scale of 1 (not helpful) to 5 (very helpful). The mean score was 4.2 (±1.0). Participants were also asked what they found clear or unclear in the questionnaire, using the questions ‘What did you find clear in the questionnaire?’ and ‘What did you find unclear in the questionnaire?’ with response options including ‘questions’, ‘food portion images’, ‘text description of food portion sizes’, ‘choice of foods’ and ‘everything was unclear’. A total of 63.2% of participants reported all aspects of the PREDICT-FFQ-v1 as ‘clear’. No participants reported that ‘everything was unclear’. A total of 75.0% (*n* = 51) reported the questions themselves to be clear, 80.9% (*n* = 55) found the food portion size images clear, 66.2% (*n* = 45) reported the text description of food portion sizes to be clear, and 80.9% (*n* = 55) reported there was a clear choice of foods in the PREDICT-FFQ-v1.

### Comparison of nutrient and food group intake measured by PREDICT-FFQ-v1 and repeated 24-h recalls

**Figure 3.**
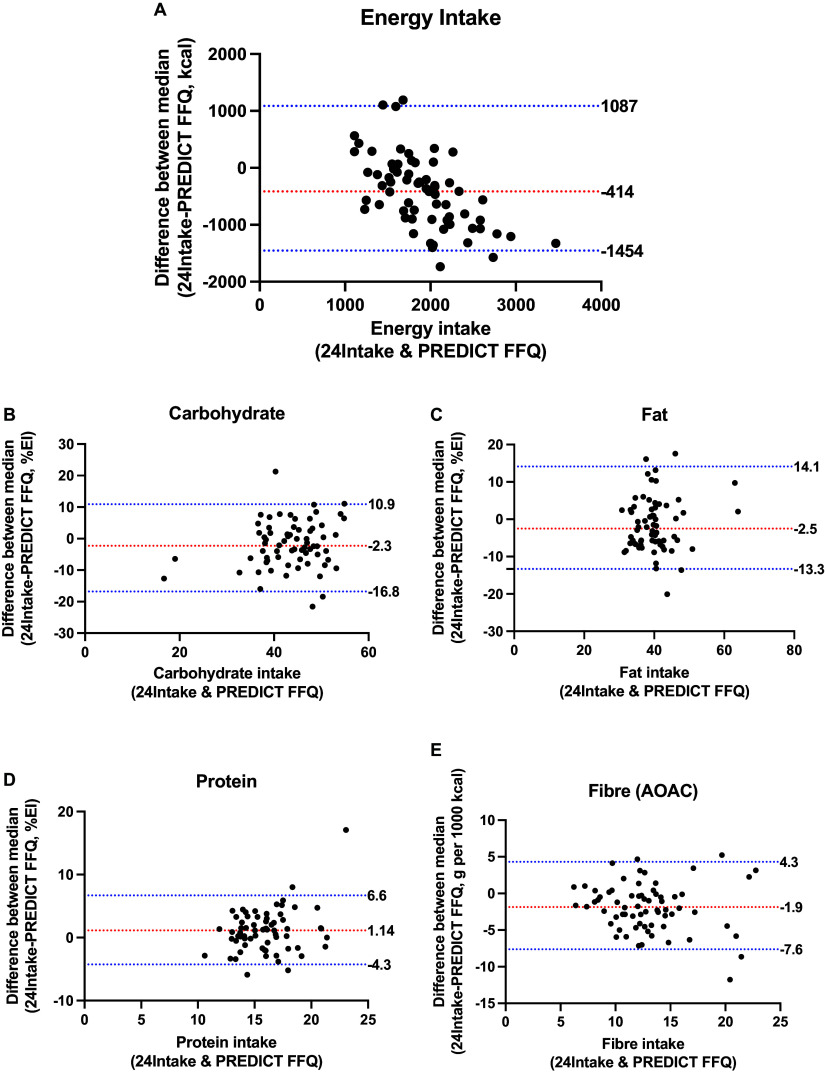
Bland–Altman plots. (A) median energy intake and energy-adjusted intakes of (B) carbohydrate (% energy intake (EI)), (C) fat (%EI), (D) protein (%EI), and (E) fibre (g per 1000 kcal) estimated from repeated 24-h dietary recalls and the PREDICT-FFQ-v1. The red dotted line shows the median difference. The blue dotted lines show the limits of agreement (2.5 and 95.7 percentiles).

Bland–Altman plots (Figure 3) illustrate the agreement (median of the differences) between the assessment tools for intakes of energy, fat, protein, carbohydrate and fibre, including 95% LOAs (i.e. 2.5th and 97.5th percentiles). Visual inspection of the Bland–Altman plot for energy intake revealed a relationship between the difference and the median for the tools, such that at larger energy intakes there is a higher energy estimation using the PREDICT-FFQ-v1 (Figure 3a). For differences between the two measures, median difference (95%CI) and limits of agreement (95% LOAs) for energy are 414kcal (95%CI 289 to 609) and −1087 to 1454 (1734 kj (95%CI 1208 to 2549) and −4547 to 6082); for carbohydrate 2.29 %EI (95%CI 0.08 to 3.68) and −10.9 to 16.8; for fat 2.51 %EI (95%CI 0.23 to 3.52) and −14.1 to 13.3; for protein −1.14 %EI (95%CI −1.64 to −0.23) and −6.60 to 4.3; and for fibre (AOAC) 1.86 g/1000kcal (95%CI 1.05 to 2.65) and −4.6 to 7.6. For energy intake, *n* = 4 (5.9%) of data fell outside of the LOAs while similar agreement was observed for differences between the tools in energy-adjusted intakes of carbohydrates (%EI), fat (%EI), protein (%EI), and fibre (AOAC, g per 1000 kcal); with between 4.4–5.9% of data falling outside of the agreement limits, respectively (Figure 3b-e).

Bland–Altman plots for total sugar, alcohol, folate, sodium (Na), iron (Fe), fruit, legumes, vegetables, meat, and nuts (unit/1000 kcal) are included in Supplementary Figure 2. Significantly higher absolute intakes (median difference) of fruit (90.1g, 95%CI 77.6 to 144; *p* < 0.001), vegetables (124.0g, 95%CI 107 to 198; *p* < 0.001), and nuts (1.74g, 95%CI 0.81 to 12.9; *p* = 0.025) were reported from the PREDICT-FFQ-v1 in comparison to 24-h dietary recall, whilst intakes of legumes (−39.8g, 95%CI −62.0 to −24.7; *p* < 0.001) and meat (−35.2g, 95%CI −82.3 to −41.6; *p* < 0.001) were significantly lower. Similar trends were observed in the energy-adjusted intakes of these foods (all *p* < 0.001) as demonstrated by visual inspection of the Bland–Altman plots (Supplementary Figure 2).

Absolute and energy-adjusted intakes of selected nutrients and food groups from the PREDICT-FFQ-v1 and the median of six repeated 24-h recalls are also presented in Table 2. There were small but significant differences between the PREDICT-FFQ-v1 and Intake24 in energy-adjusted intakes of several nutrients, whereby the PREDICT-FFQ-v1 reported higher estimates for total carbohydrate (5.73 g/1000 kcal, 95%CI 0.19 to 9.19; *p* = 0.040), fat (2.79 g/1000 kcal, 95%CI 0.26 to 3.92; *p* = 0.030), saturated fat (1.76 g/1000 kcal, 95%CI 0.73 to 2.97; *p* < 0.001), total sugar (12.0 g/1000 kcal, 95%CI 7.98 to 16.1; *p* < 0.001), fibre (1.86 g/1000 kcal, 95%CI 1.05 to 2.65; *p* < 0.001), and folate (39.7 μg/1000 kcal, 95%CI 13.6 to 44.4; *p* = 0.002), and lower estimates of protein (−2.85 g/1000 kcal, 95%CI −4.09 to −0.58; *p* = 0.010), alcohol (−0.60 g/1000 kcal, 95%CI −4.72 to −1.44; *p* < 0.001), and sodium (Na) (−2.59 g/1000 kcal, 95%CI −3.36 to −2.10); *p* < 0.001) in comparison to the 24-h dietary recalls.

**Table 2.** Absolute and energy-adjusted intakes of nutrients and food groups Table 2 long description.

	Absolute intake (unit/d)					Energy density (unit/1000 kcal)				
	PREDICT-FFQ Median (p25–p75)	Intake24 Median (p25–p75)	Median difference (95% CI)	Wilcoxon (*p*-value)	rs	PREDICT-FFQ Median (p25–p75)	Intake24 Median (p25–p75)	Median difference (95% CI)	Wilcoxon (*p*-value)	ra
Energy (kj)	8760 (6758, 11112)	7252 (5814, 7988)	1734 (1208, 2549)	<0.001	0.35					
Protein (g)	79.4 (65.5, 97.2)	68.5 (54.04, 80.18)	7.61 (5.03, 18.5)	<0.001	0.43	37.2 (34.1, 42.2)	40.4 (36.4, 44.7)	−2.85 (−4.09, −0.58)	0.01	0.44
Protein (%EI)	14.9 (13.6, 16.9)	16.14 (14.6, 17.9)	−1.14 (−1.64, −0.23)	0.01	0.44					
Fats (g)	94.8 (68.3, 119)	71.4 (58.7, 83.7)	24.6 (14.3, 31.6)	<0.001	0.3	43.8 (40.6, 49.3)	43.0 (38.3, 46.6)	2.79 (0.26, 3.92)	0.03	0.28
Fats (%EI)	39.4 (36.5, 44.4)	38.7 (34.4, 42.0)	2.51 (0.23, 3.52)	0.03	0.28					
Saturated fats (g)	34.6 (24.0, 49.3)	25.8 (20.2, 30.9)	9.63 (6.61, 13.9)	<0.001	0.41	16.7 (13.6, 19.6)	15.2 (13.0, 17.8)	1.77 (0.73, 2.97)	<0.001	0.46
Cholesterol (mg)	215 (129, 266)	225 (149, 300)	−25.3 (−50.6, 4.75)	0.10	0.42	105 (71.8, 123)	132 (91.0, 180)	−34.5 (−534, −22.9)	<0.001	0.45
Carbohydrate (g)	226 (179, 287)	174 (149, 216)	50.7 (38.9, 75.3)	<0.001	0.42	111 (103, 123)	109 (95.4, 118)	5.73 (0.19, 9.19)	0.04	0.49
Carbohydrate (%EI)	44.6 (40.9, 49.3)	43.4 (38.2, 47.3)	2.29 (0.08, 3.68)	0.04	0.49					
Total sugars (g)	108 (75.0, 142)	65.7 (53.2, 88.8)	40.4 (32.4, 51.9)	<0.001	0.37	50.3 (42.7, 63.1)	41.0 (32.2, 51.0)	12.0 (7.98, 16.1)	<0.001	0.32
Fibre (AOAC) (g)	29.0 (18.54, 36.32)	18.7 (15.2, 23.7)	7.40 (6.25, 11.6)	<0.001	0.52	13.6 (11.4, 15.8)	11.6 (9.39, 13.7)	1.86 (1.05, 2.65)	<0.001	0.65
Alcohol (g)	3.41 (0.59, 8.66)	4.11 (0.00, 17.3)	−0.16 (−7.26, −0.96)	0.01	0.78	1.39 (0.32, 3.88)	2.31 (0.00, 11.5)	−0.60 (−4.72, −1.44)	<0.001	0.82
Folate (mg)	375 (289, 480)	251 (178, 349)	1278 (78.7, 158)	<0.001	0.32	180 (149, 222)	147.2 (116, 195)	39.7 (13.6, 44.4)	0.002	0.5
Sodium (Na) (mg)	1613 (1260, 2105)	1785 (1375, 2226)	−87.3 (−323, 35.5)	0.13	0.31	798 (686, 921)	1094 (904, 1277)	−259 (−336, −210)	<0.001	0.46
Iron (Fe) (mg)	12.9 (9.07, 16.8)	10.8 (8.41, 15.8)	1.56 (−0.11, 2.96)	0.06	0.31	6.32 (5.07, 7.75)	6.34 (5.31, 8.28)	0.08 (−1.21, 0.14)	0.127	0.48
Fruit (g)	168 (77.8, 274.6)	60.2 (15.0, 125)	90.1 (77.6, 144)	<0.001	0.71	76.7 (46.1, 133)	24.9 (6.70, 54.2)	28.3 (29.6, 56.1)	<0.001	0.73
Legumes (g)	43.23 (16.9, 75.9)	82.6 (35.0, 133)	−39.8 (−62.0, −24.7)	<0.001	0.38	21.2 (7.99, 33.9)	31.8 (15.2, 59.7)	−27 (−41.8, −20.8)	<0.001	0.43
Vegetables (g)	349 (198, 448)	148 (105, 232)	124 (107, 198)	<0.001	0.4	146 (110, 216)	60.6 (40.7, 92.6)	51.7 (35.1, 72.4)	<0.001	0.5
Meats (g)	39.2 (8.83, 65.1)	83.1 (13.6, 148)	−35.2 (−82.3, −41.6)	<0.001	0.78	20.7 (4.34, 31.2)	39.7 (7.06, 62.0)	−23.8 (−52.2, −31.1)	<0.001	0.79
Nuts (g)	11.9 (2.92, 25.5)	6.67 (0.00, 21.03)	1.74 (0.81, 12.9)	0.03	0.66	6.45 (1.41, 12.5)	2.30 (0.00, 7.81)	0.35 (−0.63, 4.06)	0.001	0.66
Sugar-sweetened beverages (g)	123 (43.1, 266)	135 (18.4, 308)	0.00 (−50.0, 40.8)	0.98	0.54	62.3 (16.9, 150)	54.3 (7.05, 140)	−4.91 (−44.2, 3.25)	0.435	0.57

Data are median, 25th and 75th percentiles; %EI, percentage energy intake; rs, Spearman’s correlation coefficient; ra, energy-adjusted Spearman’s correlation coefficient.

*P < 0⋅05 and **P < 0⋅01.

The classification of subjects into the same quartile of diet quality (PDI) ranged from 30.9% (unhealthful PDI) to 44.1% (healthful PDI). Exact plus adjacent agreement ranged from 76.5% (original and healthful PDI) to 82.4% (unhealthful PDI) while the disagreement ranged from 4.4% (unhealthful PDI) to 13.2% (healthful PDI) (Supplementary Table 4).

Spearman’s correlation coefficients between estimated energy-adjusted intakes from the PREDICT-FFQ-v1 and 24-h recalls ranged from 0.28 (fat) to 0.82 (alcohol). Correlations were statistically significant for all nutrients assessed as both absolute and energy-adjusted intakes. The strongest correlations in energy-adjusted nutrient intake were observed for alcohol (*r*_s_ = 0.82), dietary fibre (*r*_s_ = 0.65), and folate (*r*_s_ = 0.50), while the weakest correlation was observed for total fat (*r*_s_ = 0.28). For food group intakes, Spearman’s correlation coefficients ranged from 0.38 (legumes) to 0.78 (meat) for absolute intakes, and 0.43 (legumes) to 0.79 (meat) for energy-adjusted intakes of food groups. Correlations were statistically significant for all foods assessed as both absolute and energy-adjusted intakes. The strongest correlations in energy-adjusted food group intake were observed for meat (*r*_s_ = 0.79), fruit (*r*_s_ = 0.73), and nuts (*r*_s_ = 0.66). The weakest correlation was observed for legumes (*r*_s_ = 0.43). All correlations fell within acceptable or strong cut-offs for dietary assessment^(32,33)^.

## Discussion

The PREDICT-FFQ-v1 encompasses 265 food and beverage items, with greater granularity of single food items, and a higher number of food items compared to previous tools, for example, the EPIC FFQ (*n* = 130). The increased granularity facilitates the estimation of intakes for more individual food items, which have been disaggregated from broader food groups. The PREDICT-FFQ-v1 captures foods and beverages commercially available and commonly consumed in the modern food environment that are often limited or absent in traditional FFQs; such as blueberries, pineapple, sweet potato and individual types of beans. Furthermore, the PREDICT-FFQ-v1 includes novel healthful food items such as fermented foods (i.e. kefir and kombucha), which are widely available in the modern food landscape and intakes of which have been shown to positively influence health^(34)^. The inclusion of additional food items may partially explain why intakes of food groups such as fruits and vegetables were significantly higher in the PREDICT-FFQ-v1 in comparison to the 24-h dietary recall. Previous validation studies involving FFQs have also observed higher intakes or overestimates of similar food groups^(35)^. Many available FFQs have focused on discretionary food items, with a lower resolution on fruits and vegetables and an ultimately larger focus on energy intake. Therefore, the PREDICT-FFQ-v1 with its increased granularity of single food items and in particular greater resolution of healthful food items such as fruits and vegetables may aid research in understanding how specific foods are associated with health outcomes.

The results demonstrate that the PREDICT-FFQ-v1 estimates energy, food, and nutrient intake at levels modestly correlated with the reference tool, multiple 24-h dietary recalls. A relationship was observed between the difference and the median for energy intake, which may be explained by the increased number of food items included in the PREDICT-FFQ-v1. The increase in individual food items hold value for research in that single items can differentiate between similar foods that have divergent health effects, for example, full versus low-fat versions of the same food, and also enables calculation of diet diversity metrics shown to be associated with health, such as number of plants.^(36)^ In addition, the increased granularity of individual food items supports a food first approach to examining dietary intake, acknowledging that the composition of foods is more nuanced than their nutrient composition, such that the food matrix^(37,38)^ processing level of foods^(39)^ and the large variability of bioactive active compounds, fibres, and other chemicals in foods can impact health. Inclusion of cross-check questions in FFQs is a way to provide an additional source of information on food group intake to mitigate concerns of mis-reporting. For example, questions in the form of ‘How many servings of fruit and fruit containing dishes do you eat per week?’ can be compared with FFQ data and the frequency of intake can be adjusted accordingly^(40)^. We have added cross-check questions to the PREDICT-FFQ to enable the examination of the effect of adjusted food frequencies on estimated nutrient and food group intake. We believe this is a valuable addition to this tool for future research.

Mean energy and macronutrient intake in the UK is reported via the NDNS, which measures intake using a 4-d diet diary in a nationally representative UK adult population (aged 1.5 years and over)^(41)^. Intakes reported via the NDNS for adults aged 19 years and over (year 9–11 (2016/17–2018/19)) were similar to those derived from the PREDICT-FFQ-v1 for protein (71.5 g/d vs 79.4 g/d), and total carbohydrate (206.0 g/d vs 226.0 g/d) but lower for energy (1734 kcal (7255 kj) vs 2984 kcal (8762 kj)) and total fat (66.6 g/d vs 94.8 g/d) suggesting the PREDICT-FFQ-v1 captures differing intakes to this nationally representative population which may be explained by differences in the populations themselves, or the different tools used to collect and estimate the dietary intakes. However, participants in this study were predominantly female (75.0%), and participants were recruited from a cohort of individuals that inherently possessed a strong interest in nutrition and therefore may have had relatively similar and more health-promoting nutritional behaviours without reflection of the wider range of behaviours present in the general UK population. Intakes derived from the NDNS for both males and females were lower when compared to intakes derived from the PREDICT-FFQ-v1 for energy (males: 1944kcal (8134 kj) vs 2736 kcal (9941 kj); females: 1541kcal (6447 kj) vs 2008 kcal (8402 kj)), and similarly NDNS intakes for both males and females were lower for protein (males: 79.9g/d vs 88.3g/d; females: 63.7 g/d vs 76.5 g/d), fat (males 73.6 g/d vs 108.3 g/d; females: 66.0 g/d vs 89.4 g/d), and carbohydrate (males: 230.0 g/d vs 261.9 g/d; females: 185.0 g/d vs 229.5g/d) in comparison to those derived from the PREDICT-FFQ-v1.

Traditionally, 24-h dietary recall tools require a skilled researcher to administer the questionnaire over the phone or in person, posing a considerable burden to both researcher and respondent. The introduction of online 24-h dietary recall tools has enabled respondents to complete dietary assessment remotely, requiring minimal researcher input during the data input and nutrient calculation stages. However, there is still a requirement for multiple completions by the respondent to account for inter-day variability and this therefore poses a notable user burden. FFQs provide a suitably accurate method for dietary evaluation through remote assessment. The additional feedback provided by participants in this study in relation to their experience in completing the PREDICT-FFQ-v1 showed a high acceptability, and it only took participants on average 22 minutes to complete, despite inclusion of a much larger set of food items. This is particularly advantageous given the burdensome nature of some dietary assessment tools. The majority of participants found all aspects of the FFQ to be clear and found the addition of the images to be helpful in estimating their food intake, with food photographs depicted in standardised portion sizes previously shown to improve the accuracy of food quantification^(42)^.

Findings from a recent meta-analysis demonstrate that overall dietary intake in epidemiological settings as assessed by FFQ is comparable to that reported by between 1 and 3 d of 24-h dietary recalls^(43)^ (*r* range = 0.22–0.77; *r* median = 0.42), in alignment with overall findings in the current study. While FFQs may be somewhat cognitively demanding of the participant in their requirement to remember foods and portions consumed less frequently, they are practical and cost-effective for large epidemiological studies. The tool can be administered as a remotely completed single survey with minimal researcher effort and a one-time burden to the participant. FFQs offer the capability for large-scale, population-based dietary assessment at minimal effort to both parties with acceptable limits to accuracy. However, their reliance on long-term memory and their structure for reporting ‘usual’ intake may introduce measurement errors due to recall bias and the challenge of accurately capturing episodically consumed foods must be considered by researchers. The dietary timeframe captured by the 24-h dietary recall is relatively short-term and is user-context-specific; that is, it is sensitive to multiple forces shaping dietary quality that may differ between different periods of 24-h and is less likely to represent accurate intakes of less frequently consumed foods, for example, tree nuts, oily fish, seasonal fruits and vegetables. The FFQ captures a more continuous, longer-term, and habitual picture of an individual’s diet, and this may explain differences seen in the analyses presented here in the intakes of food groups such as vegetables and legumes. The absolute intakes of legumes reported by the PREDICT-FFQ-v1 presented here are similar to UK reported intakes as detailed in a 2022 country specific analysis^(44)^.

Correlations for nutrient intakes between the test and reference methods were strengthened when intakes were adjusted for energy, as reported previously in dietary assessment studies involving FFQs and dietary recalls^(9,45)^. Previous studies comparing FFQs with 24-h dietary recalls report similar correlations between test and reference methods^(9,46)^ and correlation ranges for energy and nutrient intakes^(45,47)^. This includes the validation of the EPIC FFQ with twelve 24-h dietary recalls^(10)^, which found similar correlations for alcohol, fibre, and carbohydrate as reported in the current study. Median energy intakes were higher when estimated from the PREDICT-FFQ-v1 in comparison to Intake24, a trend commonly observed in previous reports comparing FFQs with 24-h dietary recalls, including within the Hordaland Health Study which compared a web-based FFQ with three 24-h dietary recall interviews^(45)^, and a study conducted by Medin et al^(47)^ assessing the validity of the same WebFFQ in a different population using four 24-h dietary recalls. The magnitude of overestimation was similar to or less than previous studies^(9,45)^. Notably, 24-h dietary recall tools have been reported to underestimate energy intake^(47)^ when compared to doubly labelled water, a gold-standard reference method of dietary assessment, which may contribute to the disparity seen between the tools compared in this study. Further, the PREDICT-FFQ-v1 asks additional information on some food groups including intakes of more foods and frequency of intake across multiple situations. For example, several milk types may be consumed as drinks, in cereals or in tea and coffees and intakes of several oil types is asked. This is different from the Intake24 and may explain some of the discordance observed in the energy and fat intake between tools (Supplementary Table 5).

A strength of this study is the similarity of the online reporting format used by both assessment tools, without study staff involvement beyond receiving reminders via email to complete these tasks. This similarity of reporting format allowed a fair comparison of the tool itself without adding social bias (as observed during interviewer-administered dietary assessments) as a confounding factor. A limitation of this study is the self-reporting of anthropometric values that were used to determine BMR; this may have prevented the correct identification of misreporters of energy intake in the statistical screening process. The PREDICT-FFQ-v1 has been developed through dietary assessment of participants taking part in the PREDICT-1 and PREDICT-2 studies as well as the PREDICT-3 study, in which participants were enrolled in the commercial ZOE testing programme. Participants in the PREDICT 3 study likely have a diet that is similar to that consumed by participants in this study. The PREDICT-FFQ-v1 was developed as a single questionnaire incorporating both UK and US food items and descriptions. This could be considered a limitation due to potential discrepancies between UK and US food databases; however it could be argued that there are many similarities amongst the majority of foods consumed between the US and UK.

Further testing of the PREDICT-FFQ-v1 in more diverse and younger and older populations, as well as across seasons is necessary to determine its wider validity and generalisability of results. As there is no true gold standard that can be used to validate all of the nutrient components of a FFQ, comparison against recovery biomarkers including doubly labelled water and urinary nitrogen are required as a next step for an unbiased ascertainment of energy expenditure and protein intake. An increased sample size would facilitate subgroup analyses to examine the impact of person-specific factors, including age, sex, and BMI, on the reporting of dietary intakes. Assessing relative validity against a multiple-pass 24-h dietary recall method may introduce errors relating to the latter method such as omissions and portion size errors. Therefore, under-reporting in the recall method should be considered when interpreting the cross-comparison results. Further, Bland–Altman plots are an appropriate approach to compare measurements by two dietary collection methods, while correlations measure the strength of a relation between two variables, not the agreement between them so results should be interpreted accordingly^(29)^. There was no washout period between the two tools, necessary to reduce carryover effects of the first dietary assessment on the subsequent one. Finally, the sample size was lower than originally desired and should be considered when interpreting the results.

In summary, a new, web-based self-administered FFQ (PREDICT-FFQ-v1) has been developed, and the results presented here indicate acceptable relative validity of this tool, in comparison to another widely used dietary assessment tool (Intake24). Use of the PREDICT-FFQ-v1 tool may therefore improve dietary assessment in large cohorts for which assessment of dietary intake by a weighed food diary is particularly burdensome. The PREDICT-FFQ-v1 captures intakes of foods that have become more commonly consumed in modern diets, and with greater resolution at the food level. Overall, this facilitates a greater focus on monitoring the impact of foods and food groups on health, in comparison to a more traditional nutrient-focused approach.

## Supporting information

10.1017/jns.2026.10080.sm001 Bermingham et al. supplementary material 1 Bermingham et al. supplementary material

10.1017/jns.2026.10080.sm002 Bermingham et al. supplementary material 2 Bermingham et al. supplementary material

10.1017/jns.2026.10080.sm003 Bermingham et al. supplementary material 3 Bermingham et al. supplementary material

10.1017/jns.2026.10080.sm004 Bermingham et al. supplementary material 4 Bermingham et al. supplementary material

10.1017/jns.2026.10080.sm005 Bermingham et al. supplementary material 5 Bermingham et al. supplementary material

## Supplementary material

The supplementary material for this article can be found at https://doi.org/10.1017/jns.2026.10080.

## Figure 1. Long description

The timeline illustrates the design of a study involving dietary intake assessment. The study begins with an enrollment phase, followed by a reporting period 1 that spans from day 2 to day 4, assessing dietary intake from day 1 to day 3. This is followed by a washout period of at least 14 days. Reporting period 2 occurs from day 20 to day 22, assessing dietary intake from day 19 to day 21. Each reporting period includes two weekdays and one weekend day. The food frequency questionnaire (FFQ) is administered on day 23 to assess dietary intake over the past month. The entire study duration is at least 21 days.

Navigate back to Figure 1..

## Table 1. Long description

The table presents characteristics of 68 study participants, divided into total cohort, males, and females. It includes data on age, height, weight, BMI, physical activity, smoking status, ethnicity, education level, and vegetarian-style dietary pattern. The total cohort has an average age of 48.3 years, height of 170.1 centimeters, weight of 74.6 kilograms, and BMI of 25.7 kilograms per square meter. Males are 17 and females are 51. Males have an average age of 48.7 years, height of 178.6 centimeters, weight of 82.7 kilograms, and BMI of 25.8 kilograms per square meter. Females have an average age of 48.2 years, height of 167.3 centimeters, weight of 71.9 kilograms, and BMI of 25.7 kilograms per square meter. Physical activity is higher in males with 227.7 minutes per week compared to 163.5 minutes per week in females. Smoking status shows that 55.9 percentage of the total cohort never smoked, 42.6 percentage used to smoke, and 1.4 percentage currently smoke. Ethnicity data indicates that 91.2 percentage of the total cohort is white, 7.4 percentage is Asian or Asian British, and 1.4 percentage falls under other categories. Education level shows that 82.4 percentage have a university degree and 17.6 percentage have a non-university degree. Vegetarian-style dietary pattern is followed by 9 percentage of the total cohort.

Navigate back to Table 1..

## Table 2. Long description

The table presents a comparison of absolute and energy-adjusted intakes of selected nutrients and food groups from the PREDICT-FFQ-v1 and the mean of six repeated 24-h recalls. It includes data on energy, protein, fats, saturated fats, cholesterol, carbohydrate, total sugars, fiber (AOAC), alcohol, folate, sodium (Na), iron (Fe), fruit, legumes, vegetables, meats, nuts, and sugar-sweetened beverages. The table has 21 rows and 12 columns, with columns for median intake values, median differences, Wilcoxon p-values, and Spearman’s rank correlation coefficients (rs) for both absolute intake and energy density. Notable trends include higher estimates for total carbohydrate, fat, saturated fat, total sugar, fiber, and folate reported by PREDICT-FFQ-v1 compared to Intake24, and lower estimates for protein, alcohol, and sodium. The table highlights small but significant differences between the two methods of dietary assessment.

Navigate back to Table 2..
