# Supplementary material for: The development and validation of a digital food-frequency questionnaire to assess habitual diet: The PREDICT Food Frequency Questionnaire
Source: J Nutr Sci. 2026 Jul 27;15:e66. doi: 10.1017/jns.2026.10080 (PMC13439026; doi:10.1017/jns.2026.10080)

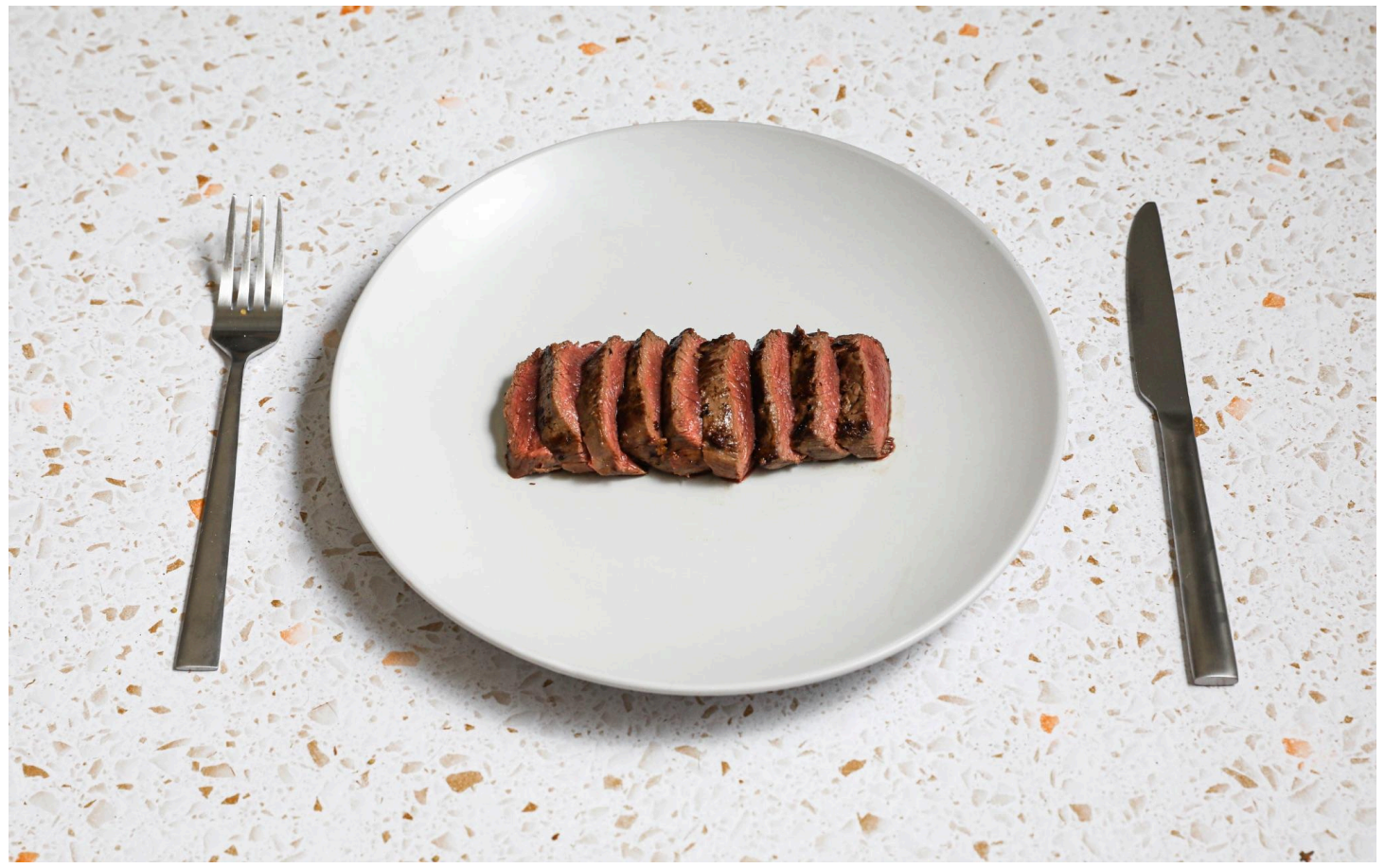
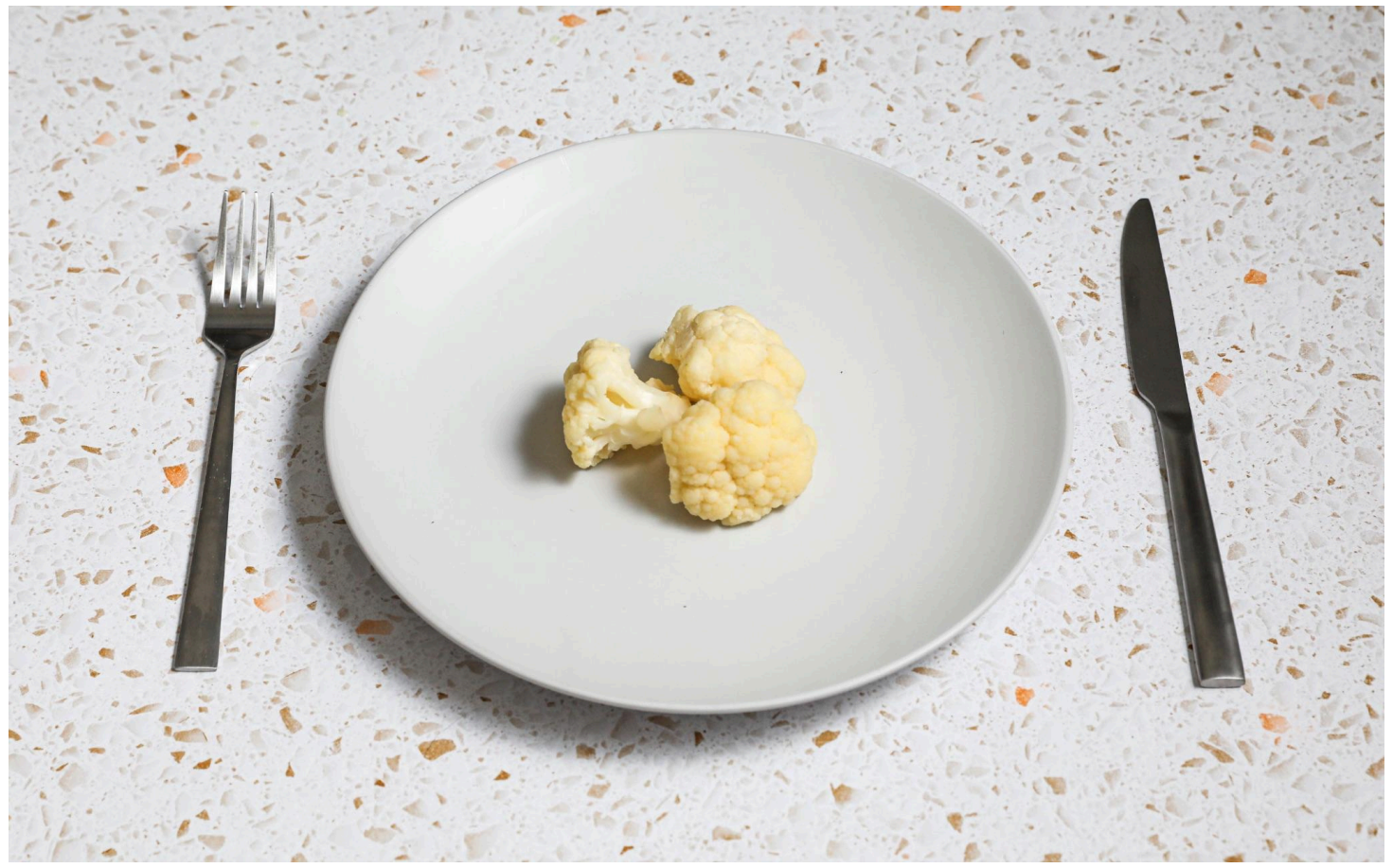
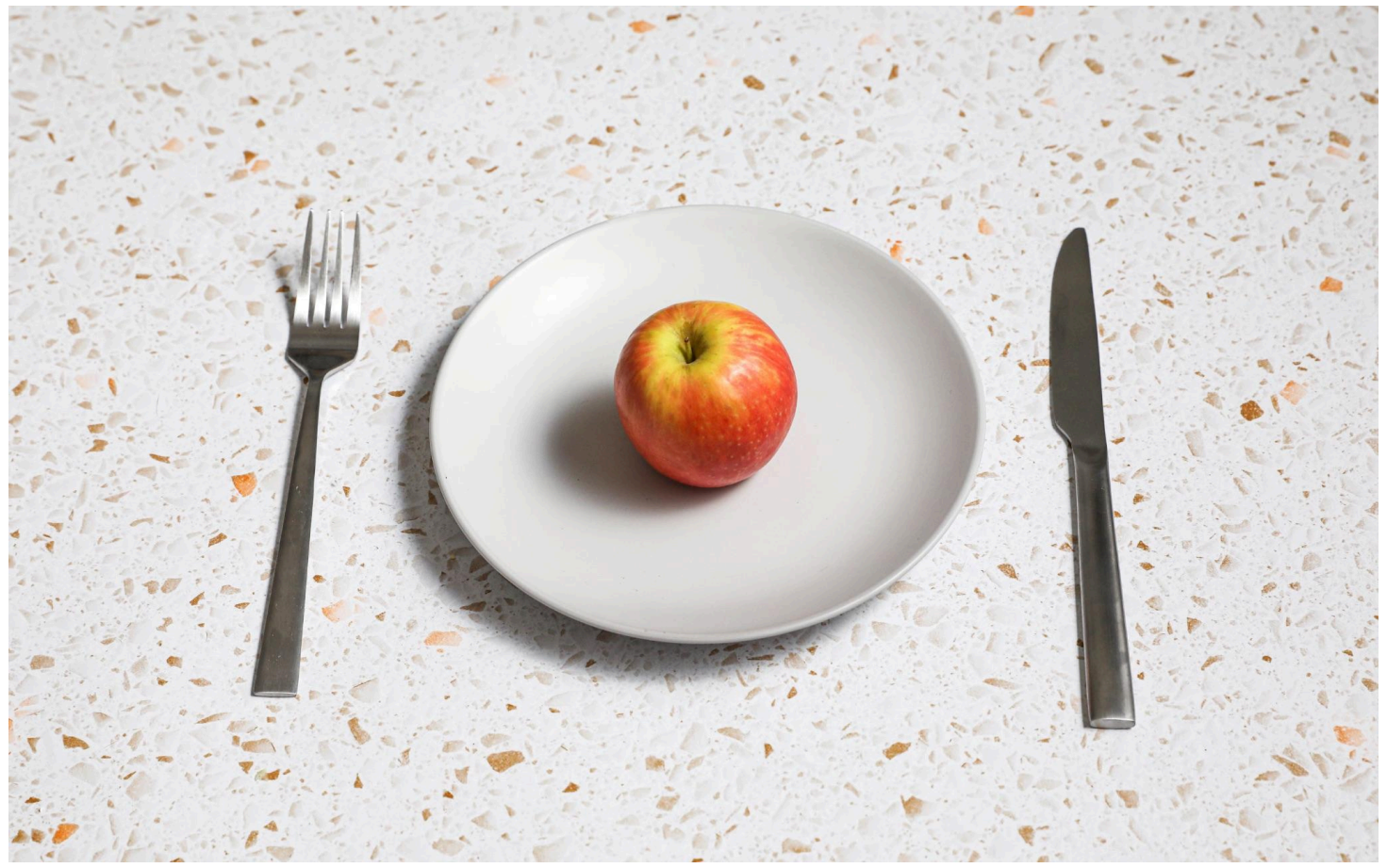
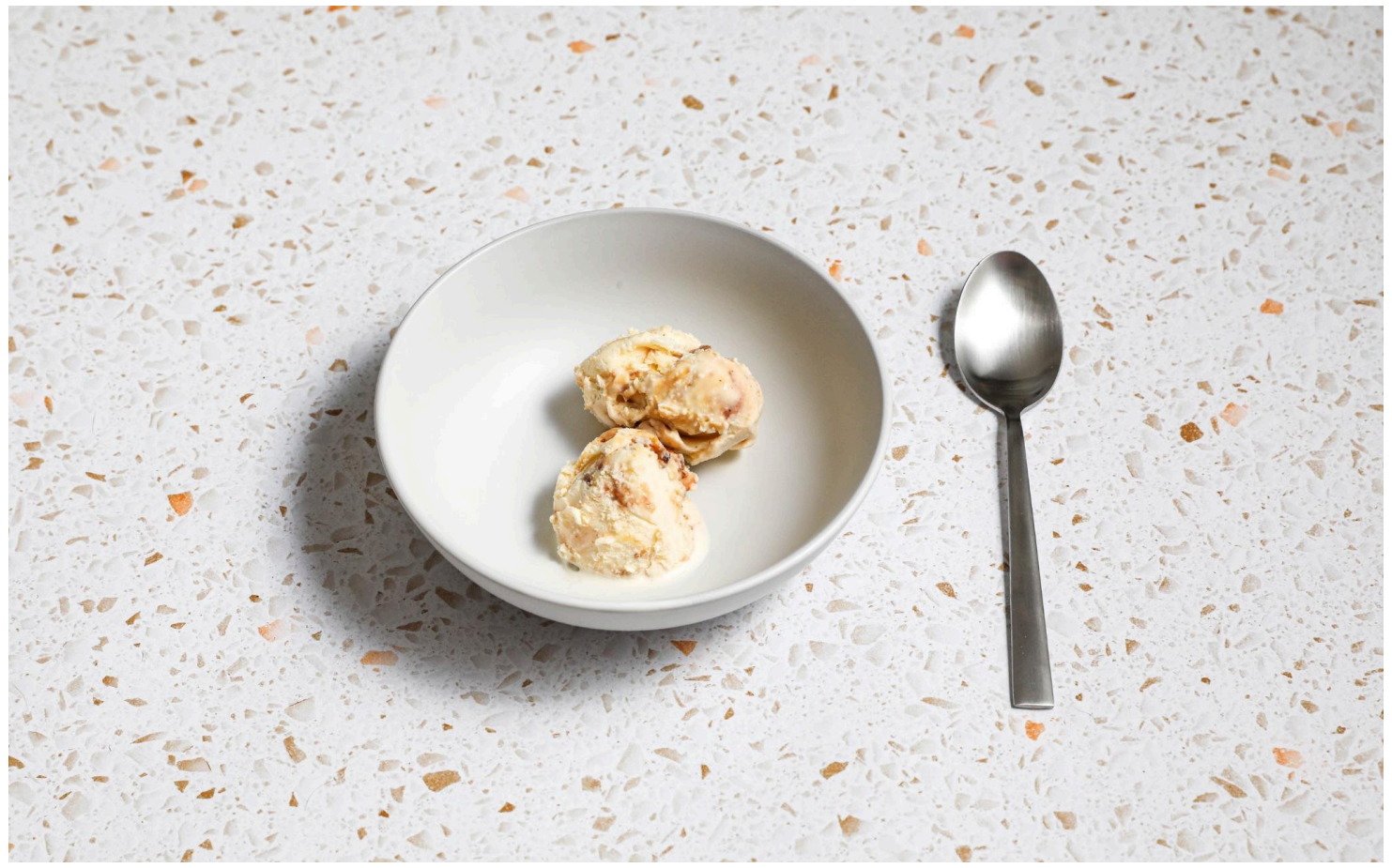

Supplement: Supplementary file 1 [file S2048679026100809sup002.pdf]
