# Supplementary material for: The development and validation of a digital food-frequency questionnaire to assess habitual diet: The PREDICT Food Frequency Questionnaire
Source: J Nutr Sci. 2026 Jul 27;15:e66. doi: 10.1017/jns.2026.10080 (PMC13439026; doi:10.1017/jns.2026.10080)
Supplement: Supplementary file 2 [file S2048679026100809sup003.pdf]

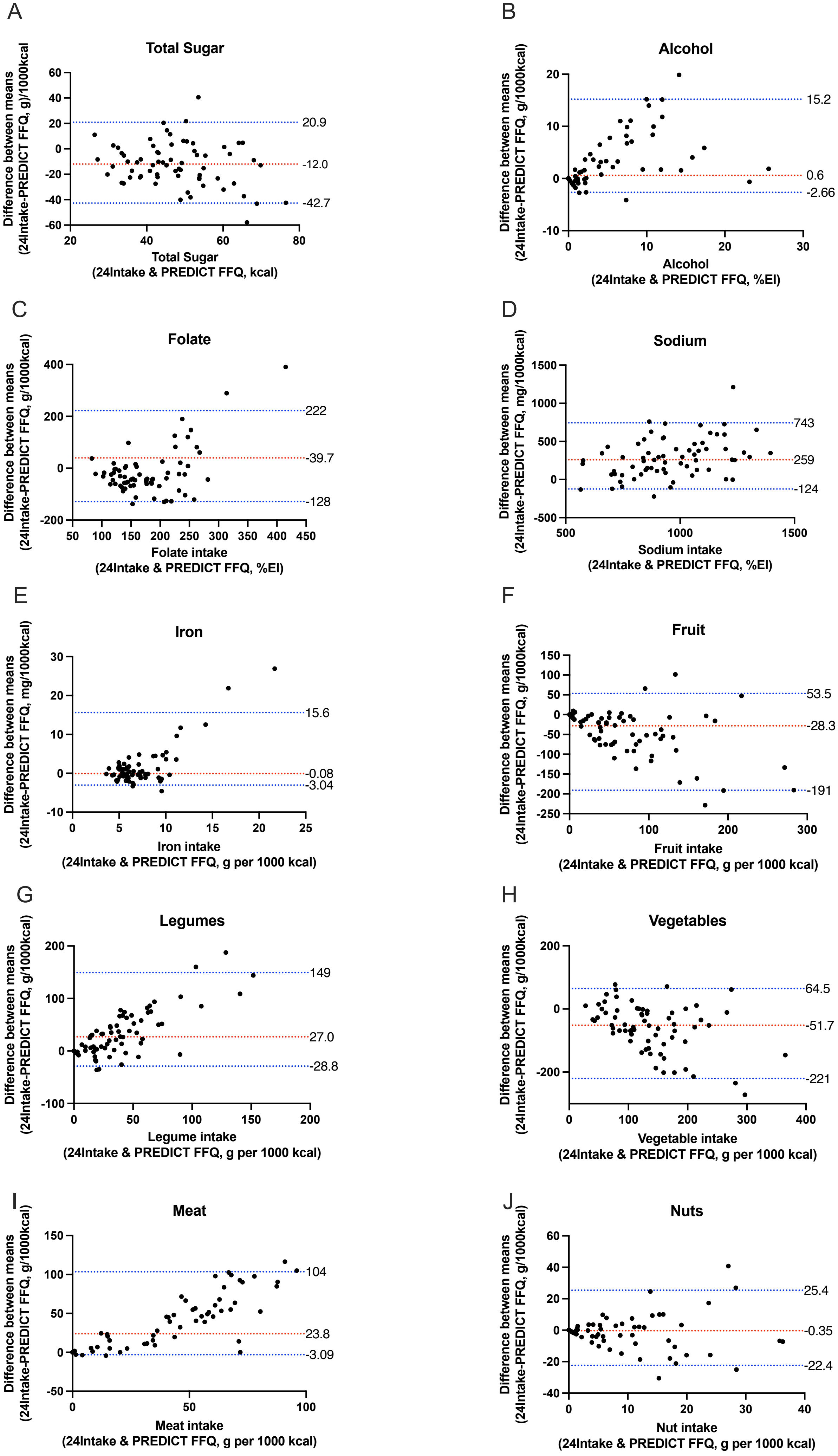
A
Total Sugar
Difference between means
(24Intake-PREDICT FFQ, g)/1000kcal
60
40
20
0
-20
-40
-60
20.9
-12.0
-42.7
20
40
60
80
Total Sugar
(24Intake & PREDICT FFQ, kcal)
B
Alcohol
Difference between means
(24Intake-PREDICT FFQ, g/1000kcal)
20
10
0
-10
15.2
0.6
-2.66
0
10
20
30
Alcohol
(24Intake & PREDICT FFQ, %EI)
C
Folate
Difference between means
(24Intake-PREDICT FFQ, g/1000kcal)
400
200
0
-200
222
-39.7
-128
50
100
150
200
250
300
350
400
450
Folate intake
(24Intake & PREDICT FFQ, %EI)
D
Sodium
Difference between means
(24Intake-PREDICT FFQ, mg/1000kcal)
1500
1000
500
0
-500
743
259
-124
500
1000
1500
Sodium intake
(24Intake & PREDICT FFQ, %EI)
E
Iron
Difference between means
(24Intake-PREDICT FFQ, mg/1000kcal)
30
20
10
0
-10
15.6
-0.08
-3.04
0
5
10
15
20
25
Iron intake
(24Intake & PREDICT FFQ, g per 1000 kcal)
F
Fruit
Difference between means
(24Intake-PREDICT FFQ, g/1000kcal)
150
100
50
0
-50
-100
-150
-200
-250
53.5
-28.3
-191
0
100
200
300
Fruit intake
(24Intake & PREDICT FFQ, g per 1000 kcal)
G
Legumes
Difference between means
(24Intake-PREDICT FFQ, g/1000kcal)
200
100
0
-100
149
27.0
-28.8
0
50
100
150
200
Legume intake
(24Intake & PREDICT FFQ, g per 1000 kcal)
H
Vegetables
Difference between means
(24Intake-PREDICT FFQ, g/1000kcal)
200
0
-200
64.5
-51.7
-221
0
100
200
300
400
Vegetable intake
(24Intake & PREDICT FFQ, g per 1000 kcal)
I
Meat
Difference between means
(24Intake-PREDICT FFQ, g/1000kcal)
150
100
50
0
-50
104
23.8
-3.09
0
50
100
Meat intake
(24Intake & PREDICT FFQ, g per 1000 kcal)
J
Nuts
Difference between means
(24Intake-PREDICT FFQ, g/1000kcal)
60
40
20
0
-20
-40
25.4
-0.35
-22.4
0
10
20
30
40
Nut intake
(24Intake & PREDICT FFQ, g per 1000 kcal)
