# Supplementary material for: The development and validation of a digital food-frequency questionnaire to assess habitual diet: The PREDICT Food Frequency Questionnaire
Source: J Nutr Sci. 2026 Jul 27;15:e66. doi: 10.1017/jns.2026.10080 (PMC13439026; doi:10.1017/jns.2026.10080)
Supplement: Supplementary file 3 [file S2048679026100809sup005.pdf]

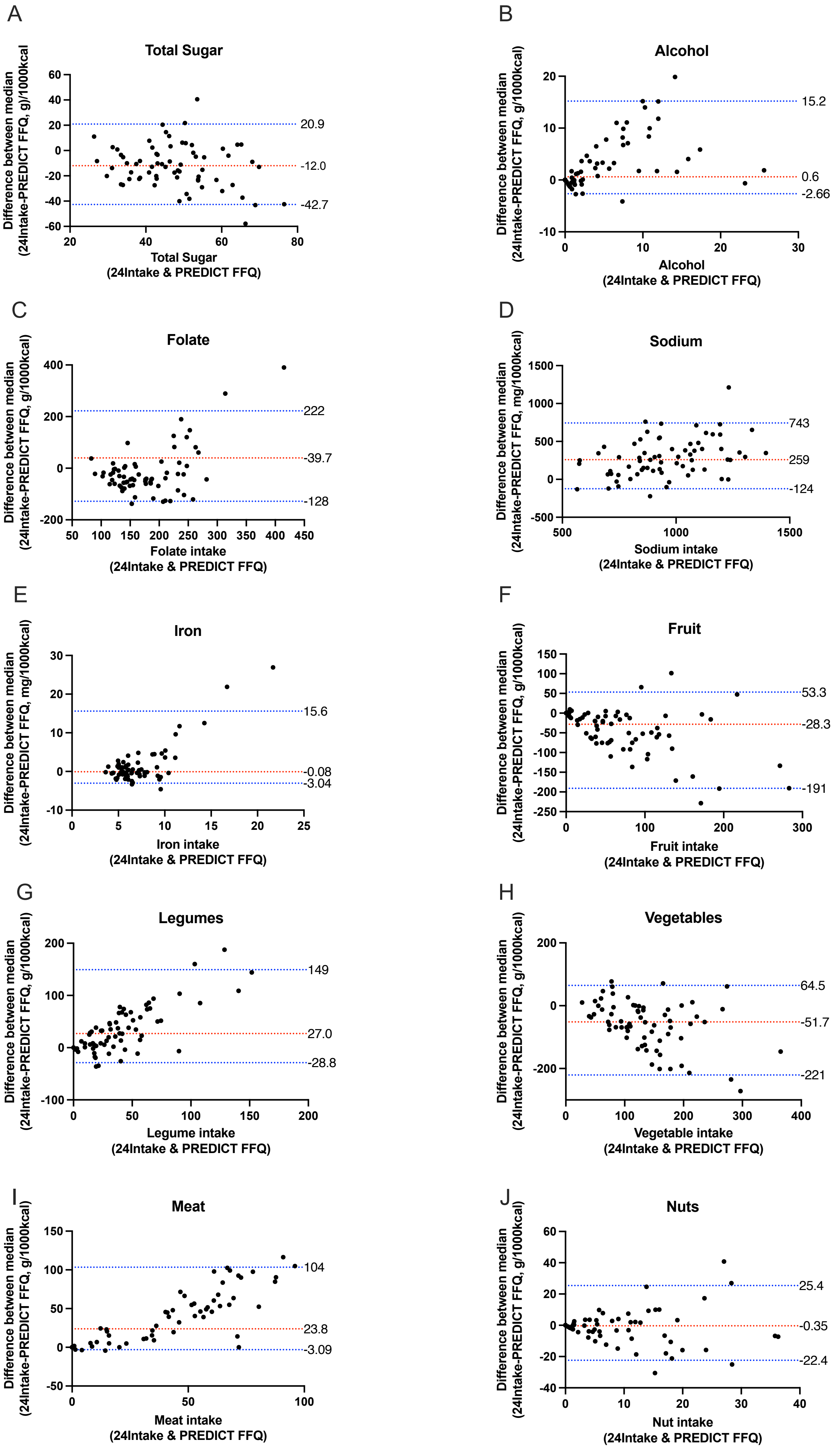

A
Total Sugar
Difference between median (24Intake-PREDICT FFQ, g)/1000kcal
Total Sugar (24Intake & PREDICT FFQ)
20.9
-12.0
-42.7
B
Alcohol
Difference between median (24Intake-PREDICT FFQ, g/1000kcal)
Alcohol (24Intake & PREDICT FFQ)
15.2
0.6
-2.66
C
Folate
Difference between median (24Intake-PREDICT FFQ, g/1000kcal)
Folate intake (24Intake & PREDICT FFQ)
222
-39.7
-128
D
Sodium
Difference between median (24Intake-PREDICT FFQ, mg/1000kcal)
Sodium intake (24Intake & PREDICT FFQ)
743
259
-124
E
Iron
Difference between median (24Intake-PREDICT FFQ, mg/1000kcal)
Iron intake (24Intake & PREDICT FFQ)
15.6
-0.08
-3.04
F
Fruit
Difference between median (24Intake-PREDICT FFQ, g/1000kcal)
Fruit intake (24Intake & PREDICT FFQ)
53.3
-28.3
-191
G
Legumes
Difference between median (24Intake-PREDICT FFQ, g/1000kcal)
Legume intake (24Intake & PREDICT FFQ)
149
27.0
-28.8
H
Vegetables
Difference between median (24Intake-PREDICT FFQ, g/1000kcal)
Vegetable intake (24Intake & PREDICT FFQ)
64.5
-51.7
-221
I
Meat
Difference between median (24Intake-PREDICT FFQ, g/1000kcal)
Meat intake (24Intake & PREDICT FFQ)
104
23.8
-3.09
J
Nuts
Difference between median (24Intake-PREDICT FFQ, g/1000kcal)
Nut intake (24Intake & PREDICT FFQ)
25.4
-0.35
-22.4
