# Supplementary material for: The development and validation of a digital food-frequency questionnaire to assess habitual diet: The PREDICT Food Frequency Questionnaire
Source: J Nutr Sci. 2026 Jul 27;15:e66. doi: 10.1017/jns.2026.10080 (PMC13439026; doi:10.1017/jns.2026.10080)
Supplement: Supplementary file 4 [file S2048679026100809sup001.docx]

**Supplementary Table 1**. Comparison of food and beverage items included in the PREDICT-FFQ-v1 and the EPIC-FFQ.

| **EPIC-FFQ** | **PREDICT-FFQ-v1** |
| --- | --- |
| Reporting period: the past year | Reporting period: the past month |
| **Meat and Fish** | |
| Average use last year - Beef: roast, steak, mince, stew or casserole | How often did you eat this amount of beef e.g. roast beef, steak, mince? |
|  | How often did you eat this amount of dried meat e.g. beef jerky, meat snacks? |
| Average use last year - Bacon | How often did you eat this amount of bacon e.g. pork bacon, turkey bacon, gammon? |
| Average use last year - Pork: roast, chops, stew or slices | How often did you eat this amount of pork e.g. roast pork, pork chops, ribs? |
| Average use last year - Lamb: roast, chops or stew | How often did you eat this amount of lamb e.g. roast lamb, loin, kofte? |
| Average use last year - Chicken or other poultry eg. turkey | How often did you eat this amount of chicken or turkey e.g. roast, baked, pieces? |
|  | How often did you eat this amount of fried, breaded or crumbed chicken e.g. pieces, nuggets, tenders? |
|  | How often did you eat this amount of duck, goose or game birds? |
| Average use last year - Ham | How often did you eat this amount of cold cuts of meat e.g. ham, corned beef, sliced meat? |
| Average use last year - Corned beef, Spam, luncheon meats |  |
| Average use last year - Sausages | How often did you eat this amount of sausage? |
| Average use last year - Beefburgers | How often did you eat this amount of beef burger in a bun? |
|  | How often did you eat this amount of beef burger without a bun? |
|  | How often did you eat this amount of hotdog or frankfurter in a bun? |
|  | How often did you eat this amount of hotdog or frankfurter without a bun? |
| Average use last year - Savoury pies, eg. meat pie, pork pie, pasties, steak & kidney pie, sausage rolls | How often did you eat this amount of savoury pie e.g. meat pie, pasties, pork pie, sausage roll? |
| Average use last year - Liver, liver paté, liver sausage | How often did you eat this amount of other meat or poultry e.g. liver, organ meat? |
| Average use last year - Oily fish, fresh or canned, eg. mackerel, kippers, tuna, salmon, sardines, herring | How often did you eat this amount of fresh salmon or trout (excluding smoked) e.g. baked, grilled, sushi? |
|  | How often did you eat this amount of smoked salmon or smoked trout? |
|  | How often did you eat this amount of other oily fish (excluding tuna and salmon) e.g. mackerel, sardines, anchovies? |
|  | How often did you eat this amount of fresh tuna? |
|  | How often did you eat this amount of swordfish? |
|  | How often did you eat this amount of other tinned fish e.g. tinned tuna, tinned salmon? |
| Average use last year - Fried fish in batter, as in fish and chips | How often did you eat this amount of fried, battered or breaded white fish? |
| Average use last year - Fish Fingers, fish cakes |  |
| Average use last year - Other white fish, fresh or frozen, eg. cod, haddock, plaice, sole, halibut | How often did you eat this amount of white fish (not fried)? |
| Average use last year - Shellfish, eg. crab, prawns, mussels | How often did you eat this amount of prawns, scampi or lobster? |
|  | How often did you eat this amount of crab? |
|  | How often did you eat this amount of mussels, oysters or clams? |
| Average use last year - Fish roe, taramasalata | How often did you eat this amount of other fish or shellfish e.g. octopus, roe, squid, fish dishes? |
|  | How often did you eat this amount of game e.g. rabbit, venison? |
| **Bread and savoury biscuits** | |
| Average use last year - Brown bread and rolls | How often did you eat this amount of wholegrain, wholemeal or brown bread e.g. sliced bread, rolls, bagel, english muffin, baguette, pitta? |
| Average use last year - Wholemeal bread and rolls | How often did you eat this amount of wholegrain, wholemeal or brown sourdough? |
| Average use last year - White bread and rolls | How often did you eat this amount of white bread e.g. sliced bread, rolls, bagel, english muffin, baguette, pitta? |
|  | How often did you eat this amount of white sourdough? |
|  | How often did you eat this amount of rye bread? |
|  | How often did you eat this amount of tortilla wrap, naan or chapati e.g. corn, wheat? |
|  | How often did you eat this amount of sandwich with meat filling e.g. ham, cold cuts of meat, beef, poultry? |
|  | How often did you eat this amount of sandwich with other filling e.g. cheese, egg, vegetables? |
|  | How often did you eat this amount of other breads e.g. crumpet, scone, cornbread muffin? |
| Average use last year - Cream crackers, cheese biscuits | How often did you eat this amount of crackers e.g. oatcakes, wholegrain crackers, water biscuits (UK) or plain crackers (US)? |
| Average use last year - Crispbread, eg. Ryvita | How often did you eat this amount of rice cake? |
|  | How often did you eat this amount of other crackers or snack products e.g. pre-packaged snack crackers, asian crackers, breadsticks? |
|  | How often did you eat this amount of gluten-free bread? |
| **Cereals** | |
| Average use last year - Porridge, Readybrek | How often did you eat this amount of cooked oatmeal or porridge? |
| Average use last year - Breakfast cereal such as cornflakes, muesli etc. | How often did you eat this amount of wholegrain breakfast cereal e.g. muesli, granola? |
|  | How often did you eat this amount of breakfast cereal e.g. plain, sugar-sweetened? |
|  | How often did you eat this amount of granola bar or cereal bar? |
|  | How often did you eat this amount of other breakfast cereals? |
| **Potatoes, rice and pasta** | |
| Average use last year - Brown rice | How often did you eat this amount of brown, wholegrain or wild rice? |
| Average use last year - White rice | How often did you eat this amount of white rice? |
| Average use last year - Wholemeal pasta | How often did you eat this amount of wholemeal pasta? |
| Average use last year - White or green pasta, eg. spaghetti, macaroni, noodles | How often did you eat this amount of pasta? |
|  | How often did you eat this amount of gluten-free pasta? |
|  | How often did you eat this amount of other grains e.g. quinoa, bulgur, farro, barley? |
|  | How often did you eat this amount of macaroni and cheese? |
|  | How often did you eat this amount of pasta salad? |
|  | How often did you eat this amount of other rice, pasta or cooked cereals e.g. noodles, polenta? |
|  | How often did you eat this amount of other grain-based dishes e.g. rice dishes, nachos, soups? |
| Average use last year - Pizza | How often did you eat this amount of pizza? |
| Average use last year - Chips | How often did you eat this amount of fried potato e.g. chips (UK) or fries (US), potato waffle, roast potato? |
| Average use last year - Roast potatoes |  |
| Average use last year - Boiled, mashed, instant or jacket potatoes | How often did you eat this amount of mashed potato? |
|  | How often did you eat this amount of other potatoes e.g. boiled, jacket potato (UK) or baked potato (US)? |
| Average use last year - Lasagne, moussaka | How often did you eat this amount of other meat-based dishes e.g. meat curry, lasagne, frozen meat-based meals? |
| Average use last year - Potato salad | How often did you eat this amount of potato salad or potato gratin? |
| **Dairy Products and fats** | |
| Average use last year - Full fat or Greek yoghurt (125g carton) | How often did you eat this amount of Greek yoghurt? |
|  | How often did you eat this amount of plain yoghurt (full fat or regular)? |
| Average use last year - Low fat yoghurt, fromage frais (125g carton) | How often did you eat this amount of plain yoghurt (low fat or fat-free)? |
|  | How often did you eat this amount of fruit yoghurt? |
|  | How often did you eat this amount of other yoghurts e.g. yoghurt drink, flavours other than fruit? |
|  | How often did you eat this amount of dairy kefir e.g. plain, flavoured? |
| Average use last year - Single or sour cream (tablespoon) | How often did you have this amount of dairy cream e.g. single cream, whipping cream, regular cream? |
| Average use last year - Double or clotted cream (tablespoon) | How often did you have this amount of sour cream? |
|  | How often did you have this amount of other dairy creams e.g. whipped cream, cream dip? |
| Average use last year - Dairy desserts (125g carton) | How often did you eat or drink this amount of other dairy desserts e.g. frozen yoghurt, milkshake, milk smoothie? |
| Average use last year - Cheese, eg. Cheddar, Brie, Edam (medium serving) | How often did you eat this amount of hard cheese e.g. cheddar, parmesan? |
|  | How often did you eat this amount of soft cheese e.g. brie, camembert? |
|  | How often did you eat this amount of mild cheese e.g. American cheese, Emmental? |
|  | How often did you eat this amount of mozzarella? |
|  | How often did you eat this amount of blue cheese e.g. danish blue, roquefort, stilton? |
|  | How often did you eat this amount of goat's or sheep's cheese e.g. feta, halloumi? |
| Average use last year - Cottage cheese, low fat soft cheese (medium serving) | How often did you eat this amount of cream cheese e.g. Philadelphia, cheese spread? |
|  | How often did you eat this amount of other cheeses e.g. ricotta, gouda, cottage cheese? |
|  | How often did you eat this amount of plant-based yoghurt? |
|  | How often did you eat this amount of plant-based cheese? |
|  | How often did you eat this amount of other dairy alternatives? |
| Average use last year - Eggs as boiled, fried, scrambled, etc. (one) | How often did you eat this amount of whole egg e.g. boiled, scrambled, fried, plain omelette? |
|  | How often did you eat this amount of egg substitute e.g. aquafaba, egg replacement? |
| Average use last year - Quiche (medium serving) | How often did you eat this amount of other egg based dishes e.g. egg white, quiche, omelette with filling? |
| Average use last year - Other salad dressing (tablespoon) | How often did you use this amount of salad dressing (regular or low fat)? |
| Average use last year - Low calorie, low fat salad cream (tablespoon) | How often did you have this amount of mayonnaise or salad cream (regular or low fat)? |
| Average use last year - Salad cream, mayonnaise (tablespoon) |  |
| **Asked in relation to bread or vegetables** | |
| Average use last year - Butter (teaspoon) | How often did you use this amount of spread e.g. margarine, olive oil spread? |
| Average use last year - Block or hard margarine, eg. Stork, Krona (teaspoon) | How often did you use this amount of other oils or spreads e.g. animal fats? |
| Average use last year - Polyunsaturated margarine, eg. Flora, sunflower, soya spreads (teaspoon) | How often did you have this amount of butter? |
| Average use last year - Soft margarines, including olive oil based and dairy spreads, eg. Blue Band, Clover (teaspoon) |  |
| Average use last year - Low fat spreads (less than 60% fat), eg.Outline, Gold(teaspoon) |  |
| Average use last year - Very low fat spread (less than 30% fat) (teaspoon) |  |
| **Sweets and snacks** | |
| Average use last year - Sweet biscuits, chocolate, eg. digestive (one) | How often did you eat this amount of biscuit, cookie or brownie? |
| Average use last year - Sweet biscuits,plain, eg. Nice,ginger (one) |  |
| Average use last year - Cakes eg. fruit, sponge, home baked | How often did you eat this amount of cake e.g. cupcake, traybake, birthday cake? |
| Average use last year - Cakes eg. fruit, sponge, readymade |  |
| Average use last year - Buns, pastries eg, scones, flapjacks, home baked | How often did you eat this amount of doughnuts or pastries? |
| Average use last year - Buns, pastries e.g.,croissants, doughnuts, ready made |  |
| Average use last year - Fruit pies, tarts, crumbles, homebaked | How often did you eat this amount of other grain desserts e.g. fruit pie, crumble? |
| Average use last year - Fruit pies, tarts, crumbles, ready made | How often did you eat this amount of pancake, waffle, eggy bread (UK) or french toast (US)? |
| Average use last year - Sponge puddings, home baked |  |
| Average use last year - Sponge puddings, readymade |  |
| Average use last year - Chocolates, single or squares | How often did you eat this amount of milk chocolate? |
| Average use last year - Chocolate snack bars eg. Mars, Crunchie | How often did you eat this amount of dark chocolate? |
|  | How often did you eat this amount of white chocolate? |
|  | How often did you eat this amount of other chocolates e.g. chocolate covered nuts, creme egg, nougat chocolate? |
| Average use last year - Sweets, toffees, mints | How often did you eat this amount of sweets e.g. fruit chews (UK) or gummies (US), marshmallow, fudge? |
|  | How often did you eat this amount of other sweets? |
|  | How often did you use this amount of syrup e.g. maple, chocolate, caramel, agave? |
| Average use last year - Sugar added to tea, coffee, cereal (teaspoon) | How often did you use this amount of added sugar e.g. in tea, coffee, cereal? |
|  | How often did you use this amount of low calorie sweetener e.g. saccharin, aspartame? |
|  | How often did you use this amount of other sugar or sweetener? |
| Average use last year - Peanuts or other nuts | How often did you eat this amount of peanuts? |
|  | How often did you eat this amount of almonds? |
|  | How often did you eat this amount of pistachio nuts? |
|  | How often did you eat this amount of walnuts? |
|  | How often did you eat this amount of cashews? |
|  | How often did you eat this amount of brazil nuts? |
|  | How often did you eat this amount of other nuts e.g. macadamia, hazelnuts, mixed nuts? |
|  | How often did you eat this amount of seeds e.g. chia, flax, sesame, pumpkin, sunflower? |
| Average use last year - Ice cream, choc ices | How often did you eat this amount of dairy ice cream? |
| Average use last year - Milk puddings, eg. rice, custard, trifle | How often did you eat this amount of custard desserts e.g. custard, creme caramel, flan? |
| Average use last year - Crisps or other packet snacks, eg. Wotsits | How often did you eat this amount of popcorn? |
|  | How often did you eat this amount of pretzels or snack mix? |
|  | How often did you eat this amount of corn snacks e.g. puffs, corn crisps (UK) or corn chips (US)? |
|  | How often did you eat this amount of potato crisps (UK) or potato chips (US)? |
| **Soups sauces and spreads** | |
| Average use last year - Peanut butter (teaspoon) | How often did you eat this amount of nut butter e.g. peanut, almond, cashew? |
| Average use last year - Meat soups (bowl) | How often did you eat this amount of meat soup e.g. meat soup with vegetables or noodles? |
| Average use last year - Sauces, eg. white sauce, cheese sauce, gravy (tablespoon) | How often did you eat this amount of other meat sauces e.g. meat gravy? |
| Average use last year - Vegetable soups (bowl) | How often did you eat this amount of vegetable soup e.g. plain, mixed, with noodles? |
|  | How often did you use two tablespoons of vinegar e.g. apple cider, wine vinegar, balsamic? |
| Average use last year - Tomato ketchup (tablespoon) | How often did you have this amount of ketchup? |
| Average use last year - Pickles, chutney (tablespoon) | How often did you have this amount of chutney or relish? |
|  | How often did you have this amount of mustard? |
|  | How often did you have this amount of other condiments e.g. BBQ sauce, soy sauce? |
| Average use last year - Jam, marmalade, honey (teaspoon) | How often did you eat this amount of jam or preserves? |
| Average use last year - Marmite, Bovril (teaspoon) | How often did you have this amount of yeast extract spread eg. marmite, vegemite? |
| **Drinks** | |
| Average use last year - Tea (cup) | How often did you drink this amount of black tea e.g. English breakfast tea, decaf black tea? |
|  | How often did you drink this amount of shop-bought sweetened iced tea? |
|  | How often did you drink this amount of herbal or fruit tea? |
|  | How often did you drink this amount of green tea? |
| Average use last year - Coffee, instant or ground (cup) | How often did you drink this amount of caffeinated coffee e.g. espresso, instant, brewed? |
| Average use last year - Coffee,decaffeinated (cup) | How often did you drink this amount of decaf coffee e.g. espresso, instant, brewed? |
|  | How often did you drink this amount of kombucha? |
|  | How often did you drink this amount of water kefir? |
| Average use last year - Low calorie or diet fizzy soft drinks (glass) | How often did you drink this amount of diet fizzy drink or diet soda? |
| Average use last year - Fizzy soft drinks, eg. Coca cola, lemonade (glass) | How often did you drink this amount of fizzy drink or soda? |
| Average use last year - Fruit squash or cordial (glass) | How often did you drink this amount of sweetened fruit drinks e.g. cordial, squash? |
|  | How often did you drink this amount of sweetened water? |
|  | How often did you drink this amount of coconut water? |
| Average use last year - Pure fruit juice (100%) eg. orange, apple juice (glass) | How often did you drink this amount of orange or grapefruit juice? |
|  | How often did you drink this amount of apple juice? |
|  | How often did you drink this amount of vegetable juice (including tomato)? |
|  | How often did you drink this amount of fruit smoothie? |
|  | How often did you drink this amount of other juices e.g. grape, cranberry, pineapple? |
|  | How often did you drink this amount of sports or energy drink? |
|  | How often did you drink this amount of dairy-based protein shake or dairy meal replacement drink? |
|  | How often did you drink this amount of non-dairy protein shake or non-dairy meal replacement drink? |
|  | How often did you drink this amount of other beverages e.g. grain drinks, non-alcoholic alternatives? |
| Average use last year - Beer, lager or cider (half pint) | How often did you drink this amount of regular or light alcoholic beer? |
|  | How often did you drink this amount of alcoholic cider? |
| Average use last year - Wine (glass) | How often did you drink this amount of red wine? |
|  | How often did you drink this amount of white wine or champagne? |
|  | How often did you drink this amount of rose wine? |
| Average use last year - Spirits, eg. gin, brandy, whisky, vodka(single) | How often did you drink this amount of white spirits e.g. gin, vodka? |
|  | How often did you drink this amount of dark spirits e.g. dark rum, whiskey? |
| Average use last year - Port, sherry, vermouth, liqueurs (glass) | How often did you drink this amount of other alcohol e.g. cocktails, other alcoholic drinks? |
| Average use last year - Cocoa, hot chocolate(cup) | How often did you have this amount of flavoured dairy milk drinks e.g. hot chocolate, chocolate milk, strawberry milk? |
| Average use last year - Coffee whitener, eg. Coffee-mate (teaspoon) |  |
| Average use last year - Horlicks, Ovaltine (cup) |  |
| **Fruit** | |
| Average use last year - Oranges, satsumas, mandarins 1( fruit) | How often did you eat this amount of orange, clementine, satsuma, or tangerine? |
| Average use last year - Grapefruit (half) | How often did you eat this amount of grapefruit? |
| Average use last year - Dried fruit, eg. raisins, prunes (medium serving) | How often did you eat this amount of dried fruit e.g. raisins, fruit in muesli? |
|  | How often did you eat this amount of prunes? |
|  | How often did you eat this amount of dates? |
| Average use last year - Average use last year - Apples 1( fruit) | How often did you eat this amount of apple e.g. fresh apple, apple sauce, cooked apple? |
|  | How often did you eat this amount of blueberries? |
| Average use last year - Strawberries, raspberries, kiwi fruit (medium serving) | How often did you eat this amount of strawberries? |
|  | How often did you eat this amount of raspberries? |
|  | How often did you eat this amount of kiwi? |
| Average use last year - Bananas (1 fruit) | How often did you eat this amount of banana? |
| Average use last year - Grapes (medium serving) | How often did you eat this amount of grapes? |
|  | How often did you eat this amount of pineapple? |
| Average use last year - Pears (1 fruit) | How often did you eat this amount of pear? |
| Average use last year - Melon (1 slice) | How often did you eat this amount of melon e.g. honeydew, cantaloupe, watermelon? |
| Average use last year - Peaches, plums, apricots (1 fruit) | How often did you eat this amount of peach? |
|  | How often did you eat this amount of plum? |
|  | How often did you eat this amount of apricot? |
|  | How often did you eat this amount of nectarine? |
|  | How often did you eat this amount of blackberries? |
|  | How often did you eat this amount of cherries? |
|  | How often did you eat this amount of mango? |
|  | How often did you eat this amount of pomegranate? |
| Average use last year - Tinned fruit (medium serving) | How often did you eat this amount of other fruit e.g. fruit salad, mixed fruit, other fruits? |
| **Vegetables** | |
| Average use last year - Avocado | How often did you eat this amount of avocado or guacamole? |
| Average use last year - Broccoli,spring greens, kale | How often did you eat this amount of broccoli? |
|  | How often did you eat this amount of kale? |
| Average use last year - Spinach | How often did you eat this amount of spinach? |
| Average use last year - Green beans, broadbeans, runner beans | How often did you eat this amount of green beans or runner beans? |
| Average use last year - Watercress | How often did you eat this amount of watercress, rocket or arugula? |
| Average use last year - Carrots | How often did you eat this amount of carrot? |
|  | How often did you eat this amount of sweet potato? |
|  | How often did you eat this amount of squash or pumpkin? |
| Average use last year - Tomatoes | How often did you eat this amount of tomato e.g. raw, cooked, salsa? |
| Average use last year - Onions | How often did you eat this amount of onion? |
| Average use last year - Garlic | How often did you eat this amount of garlic? |
|  | How often did you eat this amount of radish? |
| Average use last year - Green salad, lettuce, cucumber,celery | How often did you eat this amount of lettuce e.g. both light and dark varieties? |
|  | How often did you eat this amount of cucumber? |
|  | How often did you eat this amount of celery? |
| Average use last year - Cauliflower | How often did you eat this amount of cauliflower? |
|  | How often did you eat this amount of homemade or store-bought tomato sauce, tinned tomatoes or passata? |
| Average use last year - Sweet peppers | How often did you eat this amount of bell pepper or capsicum? |
| Average use last year - Cabbage | How often did you eat this amount of cabbage? |
| Average use last year - Peas | How often did you eat this amount of peas e.g. green peas, sugar snap? |
| Average use last year - Mushrooms | How often did you eat this amount of mushroom? |
| Average use last year - Marrow, courgettes | How often did you eat this amount of courgette (UK) or zucchini (US) or marrow? |
| Average use last year - Leeks | How often did you eat this amount of leeks? |
| Average use last year - Beetroot | How often did you eat this amount of beetroot? |
| Average use last year - Brussels sprouts | How often did you eat this amount of brussel sprouts? |
| Average use last year - Beansprouts | How often did you eat this amount of sprouts e.g. beansprouts, broccoli sprouts, lentil sprouts? |
| Average use last year - Sweetcorn | How often did you eat this amount of sweetcorn e.g. on the cob, tinned, frozen? |
|  | How often did you eat this amount of asparagus? |
|  | How often did you eat this amount of aubergine (UK) or eggplant (US)? |
|  | How often did you eat this amount of artichoke? |
| Average use last year - Parsnips, turnips, swedes | How often did you eat this amount of parsnip? |
|  | How often did you eat this amount of turnip or swede? |
| Average use last year - Coleslaw | How often did you eat this amount of coleslaw? |
|  | How often did you eat this amount of gherkins, pickles or pickled vegetables? |
|  | How often did you eat this amount of chilli pepper? |
|  | How often did you eat this amount of herbs e.g. coriander (UK) or cilantro (US), parsley, mint, basil? |
|  | How often did you eat this amount of kimchi? |
|  | How often did you eat this amount of sauerkraut? |
|  | How often did you eat this amount of seaweed e.g. nori? |
|  | How often did you eat this amount of olives e.g. green, black? |
|  | How often did you eat this amount of other vegetable dishes e.g. vegetables with meat, fish, dairy or grains? |
|  | How often did you eat this amount of other green vegetables e.g. spring greens, cavolo nero, chard? |
|  | How often did you eat this amount of other vegetables e.g. mixed vegetables, other vegetables? |
| Average use last year - Baked beans | How often did you eat this amount of white beans e.g. cannellini beans, butter beans? |
| Average use last year - Dried lentils,beans, peas | How often did you eat this amount of black beans? |
|  | How often did you eat this amount of pinto beans? |
|  | How often did you eat this amount of kidney beans? |
|  | How often did you eat this amount of mung beans? |
|  | How often did you eat this amount of refried beans? |
|  | How often did you eat this amount of baked beans? |
|  | How often did you eat this amount of chickpeas? |
|  | How often did you eat this amount of hummus? |
|  | How often did you eat this amount of lentils? |
|  | How often did you eat this amount of miso? |
|  | How often did you eat other beans and legumes e.g. falafel, bean dishes, bean soup? |
| Average use last year - Tofu, soya meat,TVP, Vegeburger | How often did you eat this amount of meat substitutes e.g. soy burger, Quorn, veggie burger? |
|  | How often did you eat this amount of tempeh? |
|  | How often did you eat this amount of tofu? |
| **Milks** | |
| What type of milk did you most often use? (Full cream/whole/ Skimmed/ Dried/ Semi-skimmed/ Channel islands, gold/ Soya) | How often did you have this amount of dairy milk as a drink? |
| How much milk did you drink each day, including milk with tea, coffee, cereals etc? (None/ Quarter of a pint/ Half a pint/ Three quarters of a pint/ One pint/ More than one pint) | How often did you use this amount of dairy milk for cereal? |
|  | How often did you use this amount of dairy milk in tea or coffee? |
|  | How often did you have this amount of dairy creamer in tea or coffee? |
|  | How often did you use this amount of plant-based creamer or cream in tea or coffee? |
|  | How often did you use this amount of plant-based milk for cereal? |
|  | How often did you use this amount of plant-based milk in tea or coffee? |
|  | How often did you have this amount of plant-based milk to drink? |
| **Fats/Oils/Fried foods** | |
| What kind of fat did you most often use for frying, roasting, grilling etc? (Butter/ Lard/dripping/ Vegetable oil/ Solid vegetable fat/ Margarine) | How often did you use two teaspoons of olive oil (excluding extra virgin olive oil)? |
| What kind of fat did you most often use for baking cakes etc? (Butter/ Lard/dripping/ Vegetable oil/ Solid vegetable fat/ Margarine) | How often did you have two teaspoons of extra virgin olive oil? |
| How often did you eat fried food at home? (Daily/ 1-3 times a week/ 4-6 times a week/ Never/ Less than once a week) | How often did you use two teaspoons of vegetable oil? |
| How often did you eat fried food away from home? (Daily/ 1-3 times a week/ 4-6 times a week/ Never/ Less than once a week) | How often did you use two teaspoons of sunflower oil? |
| What did you do with the visible fat on your meat? (Ate some of the fat/ Ate most of the fat/ Ate as little as possible/ Did not eat meat) | How often did you use two teaspoons of coconut oil? |
| How often did you eat grilled or roast meat? | How often did you use this amount of spread e.g. margarine, olive oil spread? |
| How well cooked did you usually have grilled or roast meat? (Well done/dark brown/ Medium/ Lightly cooked/rare/ Did not eat meat ) |  |
| **Salt** | |
| How often did you add salt to food while cooking? (Always/ Usually/ Rarely/ Never/ Sometimes) |  |
| How often did you add salt to any food at the table? (Always/ Usually/ Rarely/ Never/ Sometimes) |  |
| Did you regularly use a salt substitute (eg LoSalt)? If YES, which brand? |  |
| **Other** | |
| Frequency; During the course of last year,on average, how many times a week did you eat the following foods? (Vegetables (not including potatoes)/ Salad/ Fruit and fruit products (not including fruit juice)/ Fish and fish products/ Meat, meat products and meat dishes (including bacon, ham and chicken)) | |
| Cereals; Did you usually eat breakfast cereal (excluding porridge andReady Brek mentioned earlier) If YES, which brand and type of breakfast cereal, including muesli, did you usually eat? List the one or two types most often used | |

**Supplementary Table 2.** Frequency levels in the PREDICT-FFQ-v1

| Frequency |  |
| --- | --- |
| 1 time in the past month | 1 per month |
| 2 times in the past month | twice a month |
| 2-3 times in the past month | 2.5 times a month |
| Once a week | once a week |
| 1-2 times per week | 1.5 times a week |
| 2 times a week | twice a week |
| 3-4 times a week | 3.5 times a week |
| 5-6 times a week | 5.5 times a week |
| Once a day | once a day |
| 1 time per day | once a day |
| 2 or more times a day | twice a day |
| 2-3 times per day | 2.5 times per day |
| 4-5 times per day | 4.5 times per day |
| 6 or more times per day | six times per day |

**Supplementary Table 3.** PREDICT-FFQ-v1 questions including a source of meat, poultry and seafood.

How often did you eat this amount of beef e.g. roast beef, steak, mince?

How often did you eat this amount of bacon e.g. pork bacon, turkey bacon, gammon?

How often did you eat this amount of pork e.g. roast pork, pork chops, ribs?

How often did you eat this amount of lamb e.g. roast lamb, loin, kofte?

How often did you eat this amount of chicken or turkey e.g. roast, baked, pieces?

How often did you eat this amount of fried, breaded or crumbed chicken e.g. pieces, nuggets, tenders?

How often did you eat this amount of game e.g. rabbit, venison?

How often did you eat this amount of duck, goose or game birds?

How often did you eat this amount of sausage?

How often did you eat this amount of cold cuts of meat e.g. ham, corned beef, sliced meat?

How often did you eat this amount of beef burger in a bun?

How often did you eat this amount of beef burger without a bun?

How often did you eat this amount of hotdog or frankfurter in a bun?

How often did you eat this amount of hotdog or frankfurter without a bun?

How often did you eat this amount of dried meat e.g. beef jerky, meat snacks?

How often did you eat this amount of savoury pie e.g. meat pie, pasties, pork pie, sausage roll?

How often did you eat this amount of meat soup e.g. meat soup with vegetables or noodles?

How often did you eat this amount of other meat sauces e.g. meat gravy?

How often did you eat this amount of other meat-based dishes e.g. meat curry, lasagne, frozen meat-based meals?

How often did you eat this amount of other meat or poultry e.g. liver, organ meat?

How often did you eat this amount of fresh salmon or trout (excluding smoked) e.g. baked, grilled, sushi?

How often did you eat this amount of smoked salmon or smoked trout?

How often did you eat this amount of other oily fish (excluding tuna and salmon) e.g. mackerel, sardines, anchovies?

How often did you eat this amount of other tinned fish e.g. tinned tuna, tinned salmon?

How often did you eat this amount of fresh tuna?

How often did you eat this amount of swordfish?

How often did you eat this amount of white fish (not fried)?

How often did you eat this amount of fried, battered or breaded white fish?

How often did you eat this amount of prawns, scampi or lobster?

How often did you eat this amount of crab?

How often did you eat this amount of mussels, oysters or clams?

How often did you eat this amount of other fish or shellfish e.g. octopus, roe, squid, fish dishes?

**Supplementary Table 4.** Agreement of the plant-based dietary index (PDI) scores within quartiles when calculated from the PREDICT-FFQ-v1 and Intake24

|  | Exact classification | Exact or adjacent classification | Opposite classification |
| --- | --- | --- | --- |
| Original PDI | 33.80% | 76.50% | 5.90% |
| Healthful PDI | 44.10% | 82.40% | 13.20% |
| Unhealthful PDI | 30.90% | 76.50% | 4.40% |

| **Supplementary Table 5.** Percent contribution of food groups to average daily total energy and fat intake from the NDNS (19 to 64 years) and AFFIRM (PREDICT-FFQ-v1 and Intake24) Study. | | | | | | |
| --- | --- | --- | --- | --- | --- | --- |
| Food Group | NDNS % contribution to average daily total calorie intake | NDNS % contribution to average daily total fat intake | PREDICT-FFQ % contribution to average daily total calorie intake | PREDICT-FFQ % contribution to average daily total fat intake | Intake24 % contribution to average daily total calorie intake | Intake24 % contribution to average daily total fat intake |
| Cereals and cereal products | 38 | 30 | 24 | 12 | 32 | 21 |
| Milk and milk products and alternatives | 9 | 12 | 19 | 21 | 9 | 12 |
| Eggs and egg dishes | 2 | 4 | 1 | 2 | 3 | 5 |
| Fats and oils | 2 | 6 | 10 | 23 | 4 | 9 |
| Meat and meat products | 15 | 19 | 5 | 6 | 11 | 14 |
| Fish and fish dishes | 3 | 4 | 2 | 3 | 2 | 3 |
| Vegetables and vegetable products and dishes | 9 | 9 | 13 | 10 | 11 | 10 |
| Savoury snacks | 3 | 4 | 0 | 0 | 2 | 2 |
| Nuts and seeds | 1 | 3 | 6 | 12 | 5 | 10 |
| Fruit | 4 | 1 | 7 | 3 | 4 | 2 |
| Sugar, preserves and confectionery | 5 | 4 | 7 | 8 | 5 | 5 |
| Non-alcoholic beverages | 3 | 0 | 1 | 0 | 4 | 2 |
| Alcoholic beverages | 4 | 0 | 2 | 0 | 5 | 0 |
| Miscellaneous | 3 | 4 | 1 | 1 | 4 | 5 |
| National Diet and Nutrition Survey - Results from Years 12 to 15 (2019 to 2023) age 19 to 64 years presented. The NDNS (2019 to 2023) used the Intake24 tool. | | | | | | |
