# Supplementary material for: The development and validation of a digital food-frequency questionnaire to assess habitual diet: The PREDICT Food Frequency Questionnaire
Source: J Nutr Sci. 2026 Jul 27;15:e66. doi: 10.1017/jns.2026.10080 (PMC13439026; doi:10.1017/jns.2026.10080)
Supplement: Supplementary file 5 [file S2048679026100809sup004.docx]

**PREDICT-FFQ-v1 Data Dictionary**

# *Overview*

This tool is a non-qualitative, online, food frequency questionnaire, which includes 265 food and beverage items. The aim of the tool is to measure dietary intake by capturing dietary data from the past month for population surveys (ranging from 1 time in the past month to 2 or more times a day).

The PREDICT-FFQ-v1 is delivered (and validated) completely remotely and can be completed online either by using a smartphone or computer.

The PREDICT-FFQ-v1, a self-administered, quantitative, online questionnaire was developed to capture intakes of discrete dietary items at a breadth reflective of modern diets. It captures intakes of commonly consumed foods and drinks, as well as more novel food items i.e., fermented foods, in average UK and US diets. The tool was validated in 2025.

This tool is made freely available under licence for non-commercial use under Creative Commons Attribution-NonCommercial-NoDerivatives 4.0 International.

When publishing research findings using data derived from the PREDICT-FFQ-v1, the following paper must be correctly cited:

Bermingham KM, Linenberg I, Francis L et al. (2026) The development and validation of a digital food-frequency questionnaire to assess habitual diet: The PREDICT Food Frequency Questionnaire.

Please see below a description of the PREDICT-FFQ-v1.

If you are interested in using the PREDICT-FFQ-v1 in your research please contact; data.papers@joinzoe.com

## Introduction *text* from PREDICT-FFQ-v1

**Here's a few helpful pointers before you begin.**

1. We'll ask you which foods you've eaten in the past ***month*** only.

2. There are both images and text descriptions of the amount of food to guide you.

3. We'll ask you how often you ate a particular amount of food shown in an image with a text description. If you ate less or more, **select how often you ate the set amount overall.**

For example: We'll ask you how often you ate *half* *a grapefruit*. You ate one whole grapefruit once a week. In order to report **how often you ate the set amount overall**, select "twice a week".

(one whole grapefruit once a week = half a grapefruit twice a week)

## Beef, pork, poultry and other meats

**Let's start with beef, pork, poultry and other meats.**

| **Food** | **Serving description** | **Average use over past month** | | | | | | | |
| --- | --- | --- | --- | --- | --- | --- | --- | --- | --- |
| **Please select which types of beef, pork, poultry and other meats you've eaten in the past month.**  If you didn't eat any beef, pork, poultry or other meats in the past month, select 'I didn't eat any of the above' at the bottom of the page | | 1 time in the past month | 2-3 times in the past month | Once a week | 2 times a week | 3-4 times a week | 5-6 times a week | Once a day | 2 or more times a day |
| Beef e.g. roast beef, steak, mince | one small-medium steak, four thin slices of roast beef |  |  |  |  |  |  |  |  |
| Pork e.g. roast pork, pork chops, ribs | one cup shredded pork, three thin slices of roast pork, one pork escalope, four ribs with bone |  |  |  |  |  |  |  |  |
| Lamb e.g. roast lamb, loin, kofte | three slices of roast lamb, one cutlet with bone |  |  |  |  |  |  |  |  |
| Chicken or turkey e.g. roast, baked, pieces | one small-medium chicken breast, five slices of roast turkey |  |  |  |  |  |  |  |  |
| Fried, breaded or crumbed chicken e.g. pieces, nuggets, tenders | four pieces of breaded chicken, eight chicken nuggets, small chicken kiev |  |  |  |  |  |  |  |  |
| Game e.g. rabbit, venison | one venison fillet steak |  |  |  |  |  |  |  |  |
| Duck, goose or game bird | one leg of duck, one grouse (meat only) |  |  |  |  |  |  |  |  |
| Sausage | one pork or beef sausage |  |  |  |  |  |  |  |  |
| Bacon e.g. pork bacon, turkey bacon, gammon | three rashers of streaky or back bacon |  |  |  |  |  |  |  |  |
| Cold cuts of meat e.g. ham, corned beef, sliced meat | one slice of regular ham, two slices of parma ham |  |  |  |  |  |  |  |  |
| Beef burger in a bun | one burger patty in a bun |  |  |  |  |  |  |  |  |
| Beef burger without a bun | one burger patty without a bun |  |  |  |  |  |  |  |  |
| Hotdog or frankfurter in a bun | one hotdog in a bun |  |  |  |  |  |  |  |  |
| Hotdog or frankfurter without a bun | one hotdog without a bun |  |  |  |  |  |  |  |  |
| Dried meat e.g. beef jerky, meat snacks | a third of a cup or a large handful of beef jerky |  |  |  |  |  |  |  |  |
| Savoury pie e.g. meat pie, pasties, pork pie, sausage roll | two pork pies, two meat pies, two medium sausage rolls |  |  |  |  |  |  |  |  |
| Meat soup e.g. meat soup with vegetables or noodles | one cup of meat soup |  |  |  |  |  |  |  |  |
| Other meat sauces e.g. meat gravy | two tablespoons of gravy |  |  |  |  |  |  |  |  |
| Other meat-based dishes e.g. meat curry, lasagne, frozen meat-based meals | one slice of lasagne, average portion of tikka masala |  |  |  |  |  |  |  |  |
| Other meat or poultry e.g. liver, organ meat | one slice of pate, two slices of liver |  |  |  |  |  |  |  |  |
| I didn't eat any of the above |  |  |  |  |  |  |  |  |  |

## Fish and shellfish

**Let's talk about fish and shellfish.**

| **Food** | **Serving description** | **Average use over past month** | | | | | | | |
| --- | --- | --- | --- | --- | --- | --- | --- | --- | --- |
| **Please select which types of fish and shellfish you've eaten in the past month.**  If you didn't eat any fish and shellfish in the past month, select 'I didn't eat any of the above' at the bottom of the page | | 1 time in the past month | 2-3 times in the past month | Once a week | 2 times a week | 3-4 times a week | 5-6 times a week | Once a day | 2 or more times a day |
| Fresh salmon or trout (excluding smoked) e.g. baked, grilled, sushi | one fillet of salmon or trout |  |  |  |  |  |  |  |  |
| Smoked salmon or smoked trout | one large piece of smoked salmon or smoked trout |  |  |  |  |  |  |  |  |
| Other oily fish (excluding tuna and salmon) e.g. mackerel, sardines, anchovies | one and a half tins of sardines, one small-medium smoked mackerel, two and a half small tins of anchovies |  |  |  |  |  |  |  |  |
| Fresh tuna | one tuna steak |  |  |  |  |  |  |  |  |
| Other tinned fish e.g. tinned tuna, tinned salmon | one standard tin of tuna or other tinned fish |  |  |  |  |  |  |  |  |
| Swordfish | one swordfish steak |  |  |  |  |  |  |  |  |
| White fish (not fried) | one piece of fish fillet |  |  |  |  |  |  |  |  |
| Fried, battered or breaded white fish | one piece of fried, battered or breaded white fish |  |  |  |  |  |  |  |  |
| Prawns, scampi or lobster | eight prawns, four pieces of scampi, two tablespoons of lobster |  |  |  |  |  |  |  |  |
| Crab | one tablespoon of crab |  |  |  |  |  |  |  |  |
| Mussels, oysters or clams | two tablespoons of mussels or clams, four oysters |  |  |  |  |  |  |  |  |
| Other fish or shellfish e.g. octopus, roe, squid, fish dishes | a half cup of squid rings, one average portion of roe |  |  |  |  |  |  |  |  |
| I didn't eat any of the above |  |  |  |  |  |  |  |  |  |

## Eggs and egg substitutes

**Next up, eggs and egg substitutes.**

| **Food** | **Serving description** | **Average use over past month** | | | | | | | |
| --- | --- | --- | --- | --- | --- | --- | --- | --- | --- |
| **Please select which types of eggs and egg substitutes you've eaten in the past month.**  If you didn't eat any egg or egg substitutes in the past month, select 'I didn't eat any of the above' at the bottom of the page | | 1 time in the past month | 2-3 times in the past month | Once a week | 2 times a week | 3-4 times a week | 5-6 times a week | Once a day | 2 or more times a day |
| Whole egg e.g. boiled, scrambled, fried, plain omelette | one egg |  |  |  |  |  |  |  |  |
| Egg substitute e.g. aquafaba, egg replacement | a half cup of egg replacement |  |  |  |  |  |  |  |  |
| Other egg based dishes e.g. egg white, quiche, omelette with filling | one slice of quiche, two egg omelette with filling |  |  |  |  |  |  |  |  |
| I didn't eat any of the above |  |  |  |  |  |  |  |  |  |

## Grains and potatoes

**Let's move on to grains and potatoes.**

We'll split these into four categories:

1. Bread and bread products

2. Pasta, rice and other grains

3. Potatoes

4. Breakfast cereals, snacks and sweet baked goods

### Bread and bread products

| **Food** | **Serving description** | **Average use over past month** | | | | | | | |
| --- | --- | --- | --- | --- | --- | --- | --- | --- | --- |
| **Please select which types of bread and bread products you've eaten in the past month.**  If you didn't eat any bread or bread products in the past month, select 'I didn't eat any of the above' at the bottom of the page | | 1 time in the past month | 2-3 times in the past month | Once a week | 2 times a week | 3-4 times a week | 5-6 times a week | Once a day | 2 or more times a day |
| White bread e.g. sliced bread, rolls, bagel, english muffin, baguette, pitta | two slices of white bread, one bagel, one medium pitta |  |  |  |  |  |  |  |  |
| White sourdough bread | two slices of white sourdough bread |  |  |  |  |  |  |  |  |
| Wholegrain, wholemeal or brown bread e.g. sliced bread, rolls, bagel, english muffin, baguette, pitta | two slices of brown bread, one brown bagel, one wholemeal pitta |  |  |  |  |  |  |  |  |
| Wholegrain, wholemeal or brown sourdough | two slices of brown sourdough bread |  |  |  |  |  |  |  |  |
| Rye bread | one slice of pumpernickel rye bread, two slices of regular rye bread |  |  |  |  |  |  |  |  |
| Tortilla wrap, naan or chapati e.g. corn, wheat | two tortilla wraps, one small naan, two chapati |  |  |  |  |  |  |  |  |
| Pizza | three regular slices of pizza (UK) or three small slices of pizza (US) |  |  |  |  |  |  |  |  |
| Sandwich with meat filling e.g. ham, cold cuts of meat, beef, poultry | one whole sandwich with meat filling |  |  |  |  |  |  |  |  |
| Sandwich with other filling e.g. cheese, egg, vegetables | one whole sandwich with other filling |  |  |  |  |  |  |  |  |
| Other breads e.g. crumpet, scone, cornbread muffin | one and a half crumpets, one large cornbread muffin |  |  |  |  |  |  |  |  |
| Gluten-free bread | two slices of white, brown or other gluten-free bread |  |  |  |  |  |  |  |  |
| I didn't eat any of the above |  |  |  |  |  |  |  |  |  |

### Pasta, rice and other grains

| **Food** | **Serving description** | **Average use over past month** | | | | | | | |
| --- | --- | --- | --- | --- | --- | --- | --- | --- | --- |
| **Please select which types of pasta, rice and other grains you've eaten in the past month.**  If you didn't eat any pasta, rice or other grains in the past month, select 'I didn't eat any of the above' at the bottom of the page | | 1 time in the past month | 2-3 times in the past month | Once a week | 2 times a week | 3-4 times a week | 5-6 times a week | Once a day | 2 or more times a day |
| White rice | one cup of cooked white rice |  |  |  |  |  |  |  |  |
| Brown, wholegrain or wild rice | one cup of cooked brown, wholegrain or wild rice |  |  |  |  |  |  |  |  |
| Other grains e.g. quinoa, bulgur, farro, barley | one cup of cooked quinoa, bulgar, farro or barley |  |  |  |  |  |  |  |  |
| Pasta | one and a quarter cup of cooked pasta |  |  |  |  |  |  |  |  |
| Wholemeal pasta | one and a quarter cup of cooked wholemeal pasta |  |  |  |  |  |  |  |  |
| Gluten-free pasta | one and a quarter cup of cooked gluten-free pasta |  |  |  |  |  |  |  |  |
| Macaroni and cheese | one cup of macaroni and cheese |  |  |  |  |  |  |  |  |
| Pasta salad | half a cup of pasta salad |  |  |  |  |  |  |  |  |
| Other rice, pasta or cooked cereals e.g. noodles, polenta | one and a half cups of other cooked rice, pasta or cooked cereals |  |  |  |  |  |  |  |  |
| Other grain-based dishes e.g. rice dishes, nachos, soups | two cups of other grain-based dishes |  |  |  |  |  |  |  |  |
| I didn't eat any of the above |  |  |  |  |  |  |  |  |  |

### Potatoes

**How about potatoes?**

| **Food** | **Serving description** | **Average use over past month** | | | | | | | |
| --- | --- | --- | --- | --- | --- | --- | --- | --- | --- |
| **Please select which types of potatoes you've eaten in the past month.**  If you didn't eat any potatoes in the past month, select 'I didn't eat any of the above' at the bottom of the page | | 1 time in the past month | 2-3 times in the past month | Once a week | 2 times a week | 3-4 times a week | 5-6 times a week | Once a day | 2 or more times a day |
| Fried potato e.g. chips (UK) or fries (US), potato waffle, roast potato | eight to ten chips (UK), large portion of french fries, three to four potato waffles, two medium roast potatoes |  |  |  |  |  |  |  |  |
| Mashed potato | a half cup or two scoops of mashed potato |  |  |  |  |  |  |  |  |
| Potato crisps (UK) or potato chips (US) | one medium packet of potato crisps (UK) or potato chips (US) |  |  |  |  |  |  |  |  |
| Potato salad or potato gratin | half a cup or two scoops of potato salad or potato gratin |  |  |  |  |  |  |  |  |
| Other potatoes e.g. boiled, jacket potato (UK) or baked potato (US) | three small boiled potatoes, half a jacket potato (UK) or baked potato (US) |  |  |  |  |  |  |  |  |
| I didn't eat any of the above |  |  |  |  |  |  |  |  |  |

### Breakfast cereals, snacks and sweet baked goods

| **Food** | **Serving description** | **Average use over past month** | | | | | | | |
| --- | --- | --- | --- | --- | --- | --- | --- | --- | --- |
| **Please select which types of breakfast cereals, snacks and sweet baked goods you've eaten in the past month.**  If you didn't eat any breakfast cereals, snacks or sweet baked goods in the past month, select 'I didn't eat any of the above' at the bottom of the page | | 1 time in the past month | 2-3 times in the past month | Once a week | 2 times a week | 3-4 times a week | 5-6 times a week | Once a day | 2 or more times a day |
| Breakfast cereal e.g. plain, sugar-sweetened | one cup of breakfast cereal |  |  |  |  |  |  |  |  |
| Wholegrain breakfast cereal e.g. muesli, granola | a quarter of a cup of granola or muesli |  |  |  |  |  |  |  |  |
| Cooked oatmeal or porridge | two cups of cooked oatmeal or porridge |  |  |  |  |  |  |  |  |
| Granola bar or cereal bar | half a large cereal bar or one small cereal bar |  |  |  |  |  |  |  |  |
| Other breakfast cereal | half a cup of other breakfast cereal |  |  |  |  |  |  |  |  |
| Corn snacks e.g. puffs, corn crisps (UK) or corn chips (US) | one cup of corn snacks, half a medium packet of corn crisps (UK) or corn chips (US) |  |  |  |  |  |  |  |  |
| Crackers e.g. oatcakes, wholegrain crackers, water biscuits (UK) or plain crackers (US) | three oatcakes, four water biscuits (UK) or plain crackers (US) |  |  |  |  |  |  |  |  |
| Popcorn | two cups of popped popcorn |  |  |  |  |  |  |  |  |
| Pretzels or snack mix | one cup of pretzels or snack mix |  |  |  |  |  |  |  |  |
| Rice cake | two rice cakes |  |  |  |  |  |  |  |  |
| Other crackers or snack products e.g. pre-packaged snack crackers, asian crackers, breadsticks | four breadsticks, one packet of asian crackers |  |  |  |  |  |  |  |  |
| Biscuit, cookie or brownie | two cookies or biscuits, one medium brownie |  |  |  |  |  |  |  |  |
| Cake e.g. cupcake, traybake, birthday cake | one slice of cake |  |  |  |  |  |  |  |  |
| Doughnut or pastry | one and a half doughnuts or pastries |  |  |  |  |  |  |  |  |
| Pancake, waffle, eggy bread (UK) or french toast (US) | two waffles, two slices of eggy bread (UK) or french toast (US) |  |  |  |  |  |  |  |  |
| Other grain desserts e.g. fruit pie, crumble | half a cup of fruit crumble, a slice of fruit pie |  |  |  |  |  |  |  |  |
| I didn't eat any of the above |  |  |  |  |  |  |  |  |  |

## Dairy and dairy products

**Up next is dairy and dairy products.**

We'll split these into four sections:

1. Dairy milk

2. Yoghurt and other dairy milks

3. Cheese

4. Other dairy products

### Dairy milk

| **Food** | **Serving description** | **Average use over past month** | | | | | | | |
| --- | --- | --- | --- | --- | --- | --- | --- | --- | --- |
| **Please select which types of dairy milk you've had in the past month.**  If you didn't have any dairy milk in the past month, select 'I didn't have any of the above' at the bottom of the page | | 1 time in the past month | 2-3 times in the past month | Once a week | 2 times a week | 3-4 times a week | 5-6 times a week | Once a day | 2 or more times a day |
| Dairy milk as a drink | 200ml or one cup of milk |  |  |  |  |  |  |  |  |
| Dairy milk for cereal | 100ml or half a cup of milk |  |  |  |  |  |  |  |  |
| Dairy milk in tea or coffee | 50ml or quarter of a cup of milk |  |  |  |  |  |  |  |  |
| I didn't have any of the above |  |  |  |  |  |  |  |  |  |

| **Type of dairy** | | | | | |
| --- | --- | --- | --- | --- | --- |
| **Please select one**  Skip this section if you selected “I didn't have any of the above” in the previous table. | Whole milk | Semi-skimmed milk (UK) | 2% milk (US) | 1% milk (US) | Skimmed milk (fat-free milk) |
| Please select which type of dairy milk you've had as a drink in the past month. |  |  |  |  |  |
| Please select which type of dairy milk you've had for cereal in the past month. |  |  |  |  |  |
| Please select which type of dairy milk you've had in tea or coffee in the past month. |  |  |  |  |  |

### Yoghurt and other dairy milks

| **Food** | **Serving description** | **Average use over past month** | | | | | | | |
| --- | --- | --- | --- | --- | --- | --- | --- | --- | --- |
| **Please select which types of yoghurt and other dairy milks you've had in the past month.**  If you didn't have any yoghurt or other dairy milks in the past month, select 'I didn't have any of the above' at the bottom of the page | | 1 time in the past month | 2-3 times in the past month | Once a week | 2 times a week | 3-4 times a week | 5-6 times a week | Once a day | 2 or more times a day |
| Greek yoghurt | half a cup or one individual pot of greek yoghurt |  |  |  |  |  |  |  |  |
| Plain yoghurt (full fat or regular) | half a cup or one individual pot of full fat plain yoghurt |  |  |  |  |  |  |  |  |
| Plain yoghurt (low fat or fat-free) | half a cup or one individual pot of fat-free yoghurt |  |  |  |  |  |  |  |  |
| Fruit yoghurt | half a cup or one individual pot of fruit yoghurt |  |  |  |  |  |  |  |  |
| Other yoghurts e.g. yoghurt drink, flavours other than fruit | half a cup or one individual pot of other flavoured yoghurt, just over half a standard yoghurt drink |  |  |  |  |  |  |  |  |
| Dairy kefir e.g. plain, flavoured | half a cup or one individual pot of kefir yoghurt, just over half a standard kefir yoghurt drink |  |  |  |  |  |  |  |  |
| Flavoured milk drinks e.g. hot chocolate, chocolate milk, strawberry milk | 190ml or one cup of flavoured dairy milk |  |  |  |  |  |  |  |  |
| I didn't have any of the above |  |  |  |  |  |  |  |  |  |

### Cheese

| **Food** | **Serving description** | **Average use over past month** | | | | | | | |
| --- | --- | --- | --- | --- | --- | --- | --- | --- | --- |
| **Please select which types of cheese you've eaten in the past month.**  If you didn't eat any cheese in the past month, select 'I didn't eat any of the above' at the bottom of the page | | 1 time in the past month | 2-3 times in the past month | Once a week | 2 times a week | 3-4 times a week | 5-6 times a week | Once a day | 2 or more times a day |
| Mild cheese e.g. American cheese, Emmental | three small chunks or one medium chunk of mild cheese |  |  |  |  |  |  |  |  |
| Blue cheese e.g. danish blue, roquefort, stilton | four small chunks or one medium chunk of blue cheese |  |  |  |  |  |  |  |  |
| Cream cheese e.g. Philadelphia, cheese spread | one heaped tablespoon of cream cheese |  |  |  |  |  |  |  |  |
| Goat's or sheep's cheese e.g. feta, halloumi | five cubes of feta |  |  |  |  |  |  |  |  |
| Hard cheese e.g. cheddar, parmesan | four small slices, one medium chunk or four grated tablespoons of hard cheese |  |  |  |  |  |  |  |  |
| Mozzarella | one tablespoon of mozzarella |  |  |  |  |  |  |  |  |
| Soft cow's cheese e.g. brie, camembert | four small slices or one medium chunk of brie or camembert |  |  |  |  |  |  |  |  |
| Other cheeses e.g. ricotta, gouda, cottage cheese | three small chunks or one medium chunk of gruyere, one tablespoon of cottage cheese |  |  |  |  |  |  |  |  |
| I didn't eat any of the above |  |  |  |  |  |  |  |  |  |

### Other dairy products

| **Food** | **Serving description** | **Average use over past month** | | | | | | | |
| --- | --- | --- | --- | --- | --- | --- | --- | --- | --- |
| **Please select which types of other dairy products you've had in the past month.**  If you didn't have any other dairy products in the past month, select 'I didn't have any of the above' at the bottom of the page | | 1 time in the past month | 2-3 times in the past month | Once a week | 2 times a week | 3-4 times a week | 5-6 times a week | Once a day | 2 or more times a day |
| Butter | one knob of butter or average spread on two pieces of toast |  |  |  |  |  |  |  |  |
| Dairy cream e.g. single cream, whipping cream, regular cream | one tablespoon of cream |  |  |  |  |  |  |  |  |
| Dairy creamer in tea or coffee | 50ml or a quarter of a cup of dairy creamer |  |  |  |  |  |  |  |  |
| Sour cream | two tablespoons of sour cream |  |  |  |  |  |  |  |  |
| Dairy ice cream | one average or two small scoops of dairy ice cream |  |  |  |  |  |  |  |  |
| Other dairy creams e.g. whipped cream, cream dip | one tablespoon of other dairy cream or cream dip |  |  |  |  |  |  |  |  |
| Custard desserts e.g. custard, creme caramel, flan | one pot of custard dessert or creme caramel, one slice of flan |  |  |  |  |  |  |  |  |
| Other dairy desserts e.g. frozen yogurt, milkshake, milk smoothie | 190ml of milkshake or milk smoothie |  |  |  |  |  |  |  |  |
| I didn't have any of the above |  |  |  |  |  |  |  |  |  |

## Plant-based milks and other meat and dairy alternatives

**Next, we'll ask about plant-based milks and other meat and dairy alternatives.**

### Plant-based milks

**First, let's talk about plant-based milks.**

| **Food** | **Serving description** | **Average use over past month** | | | | | | | |
| --- | --- | --- | --- | --- | --- | --- | --- | --- | --- |
| **Please select which types of plant-based milks you've had in the past month.**  If you didn't have any plant-based milks in the past month, select 'I didn't have any of the above' at the bottom of the page | | 1 time in the past month | 2-3 times in the past month | Once a week | 2 times a week | 3-4 times a week | 5-6 times a week | Once a day | 2 or more times a day |
| Plant-based creamer or cream in tea or coffee | 50ml or quarter of a cup of plant-based creamer or cream |  |  |  |  |  |  |  |  |
| Plant-based milk to drink | 200ml or one cup of plant-based milk |  |  |  |  |  |  |  |  |
| Plant-based milk for cereal | 100ml or half a cup of plant-based milk |  |  |  |  |  |  |  |  |
| Plant-based milk in tea or coffee | 50ml or quarter of a cup of plant-based milk |  |  |  |  |  |  |  |  |
| I didn't have any of the above |  |  |  |  |  |  |  |  |  |

| **Type of plant-based milks** | | | | | | |
| --- | --- | --- | --- | --- | --- | --- |
| **Please select the type you've had most often**  Skip this section if you selected “I didn't have any of the above” in the previous table. | Nut milk e.g. almond, hazelnut, walnut | Soy milk | Coconut milk | Rice milk | Oat milk | I used something else |
| Please select which type of plant-based creamer or cream you've used in tea or coffee in the past month. |  |  |  |  |  |  |
| Please select which type of plant-based milk you've had to drink in the past month. |  |  |  |  |  |  |
| Please select which type of plant-based milk you've had for cereal in the past month. |  |  |  |  |  |  |
| Please select which type of plant-based milk you've used in tea or coffee in the past month. |  |  |  |  |  |  |

### Meat and dairy alternatives

**Now we'll ask you about other meat and dairy alternatives.**

| **Food** | **Serving description** | **Average use over past month** | | | | | | | |
| --- | --- | --- | --- | --- | --- | --- | --- | --- | --- |
| **Please select which types of other meat and dairy alternatives you've eaten in the past month.**  If you didn't eat any other meat and dairy alternatives in the past month, select 'I didn't eat any of the above' at the bottom of the page | | 1 time in the past month | 2-3 times in the past month | Once a week | 2 times a week | 3-4 times a week | 5-6 times a week | Once a day | 2 or more times a day |
| Plant-based yoghurt | half a cup or one individual pot of plant-based yoghurt |  |  |  |  |  |  |  |  |
| Plant-based cheese | three small chunks or one medium chunk of non-dairy cheese |  |  |  |  |  |  |  |  |
| Other dairy alternatives | one tablespoon of plant-based cream cheese |  |  |  |  |  |  |  |  |
| Meat substitutes e.g. soy burger, Quorn, veggie burger | one veggie burger patty |  |  |  |  |  |  |  |  |
| Tempeh | five slices of tempeh |  |  |  |  |  |  |  |  |
| Tofu | three slices of tofu |  |  |  |  |  |  |  |  |
| I didn't eat any of the above |  |  |  |  |  |  |  |  |  |

## Oils, dressings and condiments

**Let's move on to oils, dressings and condiments.**

Note to reader: There are no food images for oils and vinegars. Use the text description to guide you on the amount.

| **Food** | **Serving description** | **Average use over past month** | | | | | | | |
| --- | --- | --- | --- | --- | --- | --- | --- | --- | --- |
| **Please select which types of oils, dressings and condiments you've had in the past month.**  If you didn't have any oils, dressings or condiments in the past month, select 'I didn't have any of the above' at the bottom of the page | | 1 time in the past month | 2-3 times in the past month | Once a week | 2 times a week | 3-4 times a week | 5-6 times a week | Once a day | 2 or more times a day |
| Extra virgin olive oil | two teaspoons |  |  |  |  |  |  |  |  |
| Olive oil (excluding extra virgin olive oil) | two teaspoons |  |  |  |  |  |  |  |  |
| Vegetable oil | two teaspoons |  |  |  |  |  |  |  |  |
| Sunflower oil | two teaspoons |  |  |  |  |  |  |  |  |
| Coconut oil | two teaspoons |  |  |  |  |  |  |  |  |
| Spread e.g. margarine, olive oil spread | one knob of spread or average spread on two pieces of toast |  |  |  |  |  |  |  |  |
| Yeast extract spread eg. marmite, vegemite | one teaspoon or a thick scraping of marmite or vegemite (e.g. on bread) |  |  |  |  |  |  |  |  |
| Salad dressing (regular or low fat) | three teaspoons of salad dressing |  |  |  |  |  |  |  |  |
| Mayonnaise or salad cream (regular or low fat) | one portion pack or one tablespoon of mayonnaise |  |  |  |  |  |  |  |  |
| Other oils or spreads e.g. animal fats | one knob of other spread, two teaspoons of other oil |  |  |  |  |  |  |  |  |
| Ketchup | four teaspoons or one portion pack of ketchup |  |  |  |  |  |  |  |  |
| Mustard | four teaspoons of mustard |  |  |  |  |  |  |  |  |
| Vinegar e.g. apple cider, wine vinegar, balsamic | two tablespoons |  |  |  |  |  |  |  |  |
| Chutney or relish | three teaspoons of chutney or relish |  |  |  |  |  |  |  |  |
| Other condiments e.g. BBQ sauce, soy sauce | four teaspoons or one portion pack of other sauce |  |  |  |  |  |  |  |  |
| I didn't have any of the above |  |  |  |  |  |  |  |  |  |

**Great progress, you're over halfway there!**

Your answers help us build the best picture of what you've eaten in the past month.

## Sweet treats

**It's time to talk about sweet treats.**

| **Food** | **Serving description** | **Average use over past month** | | | | | | | |
| --- | --- | --- | --- | --- | --- | --- | --- | --- | --- |
| **Please select which types of sweet treats you've eaten in the past month.**  If you didn't eat any sweet treats in the past month, select 'I didn't eat any of the above' at the bottom of the page | | 1 time in the past month | 2-3 times in the past month | Once a week | 2 times a week | 3-4 times a week | 5-6 times a week | Once a day | 2 or more times a day |
| Milk chocolate | five thin pieces or one bar of milk chocolate |  |  |  |  |  |  |  |  |
| Dark chocolate | five thin pieces or one bar of dark chocolate |  |  |  |  |  |  |  |  |
| White chocolate | two pieces of white chocolate, one packet of white chocolate buttons |  |  |  |  |  |  |  |  |
| Other chocolates e.g. chocolate covered nuts, creme egg, nougat chocolate | small handful of Maltesers, one creme egg, one nougat chocolate bar |  |  |  |  |  |  |  |  |
| Sweets e.g. fruit chews (UK) or gummies (US), marshmallow, fudge | small handful of sweets, seven jellies, eight marshmallows |  |  |  |  |  |  |  |  |
| Other sweets | small handful of other sweets |  |  |  |  |  |  |  |  |
| I didn't eat any of the above |  |  |  |  |  |  |  |  |  |

## Nuts and seeds

**Let's move on to nuts and seeds.**

| **Food** | **Serving description** | **Average use over past month** | | | | | | | |
| --- | --- | --- | --- | --- | --- | --- | --- | --- | --- |
| **Please select which types of nuts and seeds you've eaten in the past month.**  If you didn't eat any nuts and seeds in the past month, select 'I didn't eat any of the above' at the bottom of the page | | 1 time in the past month | 2-3 times in the past month | Once a week | 2 times a week | 3-4 times a week | 5-6 times a week | Once a day | 2 or more times a day |
| Peanuts | ten whole peanuts |  |  |  |  |  |  |  |  |
| Almonds | twelve whole almonds |  |  |  |  |  |  |  |  |
| Pistachio nuts | ten pistachio nuts |  |  |  |  |  |  |  |  |
| Walnuts | ten walnuts halves |  |  |  |  |  |  |  |  |
| Cashews | ten cashews |  |  |  |  |  |  |  |  |
| Brazil nuts | ten brazil nuts |  |  |  |  |  |  |  |  |
| Other nuts e.g. macadamia, hazelnuts, mixed nuts | a small handful of other nuts |  |  |  |  |  |  |  |  |
| Nut butter e.g. peanut, almond, cashew | one portion pack or one thick spread of nut butter (e.g. on bread) |  |  |  |  |  |  |  |  |
| Seeds e.g. chia, flax, sesame, pumpkin, sunflower | one tablespoon of seeds |  |  |  |  |  |  |  |  |
| I didn't eat any of the above |  |  |  |  |  |  |  |  |  |

## Sugars, sweeteners, jams and honey

**Let's move to sugars, sweeteners, jams and honey.**

| **Food** | **Serving description** | **Average use over past month** | | | | | | | |
| --- | --- | --- | --- | --- | --- | --- | --- | --- | --- |
| **Please select which types of sugars, sweeteners, jams and honey you've had in the past month.**  If you didn't have any sugars, sweeteners, jams or honey in the past month, select 'I didn't have any of the above' at the bottom of the page | | 1 time in the past month | 2-3 times in the past month | Once a week | 2 times a week | 3-4 times a week | 5-6 times a week | Once a day | 2 or more times a day |
| Added sugar e.g. in coffee, tea, cereal | one teaspoon of sugar |  |  |  |  |  |  |  |  |
| Low calorie sweetener e.g. saccharin, aspartame | one tablet |  |  |  |  |  |  |  |  |
| Honey | two teaspoons or one spread of honey (e.g. on bread) |  |  |  |  |  |  |  |  |
| Jam or preserves | two teaspoons or one thick spread of jam (e.g. on bread) |  |  |  |  |  |  |  |  |
| Syrup e.g. maple, chocolate, caramel, agave | two teaspoons of syrup |  |  |  |  |  |  |  |  |
| Other sugar or sweetener | two teaspoons of other sugar or sweetener |  |  |  |  |  |  |  |  |
| I didn't have any of the above |  |  |  |  |  |  |  |  |  |

## Beverages

**Next up is beverages.**

Earlier you already selected how often you drink a certain amount of milk in tea and coffee.

### Tea and coffee

| **Drink** | **Serving description** | **Average use over past month** | | | | | | | | |
| --- | --- | --- | --- | --- | --- | --- | --- | --- | --- | --- |
| **Please select which types of tea and coffee you've had in the past month.**  If you didn't drink any tea or coffee in the past month, select 'I didn't drink any of the above' at the bottom of the page | | 1 time in the past month | 2-3 times in the past month | 1-2 times per week | 3-4 times per week | 5-6 times per week | 1 time per day | 2-3 times per day | 4-5 times per day | 6 or more times per day |
| Black tea e.g. English breakfast tea, decaf black tea | 220ml or one cup of tea, black or before adding milk |  |  |  |  |  |  |  |  |  |
| Sweetened iced tea (shop-bought) | 375ml or one bottle of sweetened iced tea |  |  |  |  |  |  |  |  |  |
| Herbal or fruit tea | 220ml or one cup of herbal or fruit tea |  |  |  |  |  |  |  |  |  |
| Green tea | 220ml or one cup of green tea |  |  |  |  |  |  |  |  |  |
| Caffeinated coffee e.g. espresso, instant, brewed | 220ml or one cup of caffeinated coffee, black or before adding milk |  |  |  |  |  |  |  |  |  |
| Decaf coffee e.g. espresso, instant, brewed | 220ml or one cup of decaf coffee, black or before adding milk |  |  |  |  |  |  |  |  |  |
| I didn't drink any of the above |  |  |  |  |  |  |  |  |  |  |

### Other drinks

**What about other drinks?**

| **Drink** | **Serving description** | **Average use over past month** | | | | | | | | |
| --- | --- | --- | --- | --- | --- | --- | --- | --- | --- | --- |
| **Please select which types of other drinks you've drank in the past month.**  If you didn't drink any other drinks in the past month, select 'I didn't drink any of the above' at the bottom of the page | | 1 time in the past month | 2-3 times in the past month | 1-2 times per week | 3-4 times per week | 5-6 times per week | 1 time per day | 2-3 times per day | 4-5 times per day | 6 or more times per day |
| Fizzy drink or soda | 330ml or one standard can of fizzy drink or soda |  |  |  |  |  |  |  |  |  |
| Diet fizzy drink or diet soda | 330ml or one standard can of diet fizzy drink or diet soda |  |  |  |  |  |  |  |  |  |
| Apple juice | 220ml of apple juice |  |  |  |  |  |  |  |  |  |
| Orange or grapefruit juice | 220ml of orange juice |  |  |  |  |  |  |  |  |  |
| Fruit smoothie | 220ml of fruit smoothie |  |  |  |  |  |  |  |  |  |
| Vegetable juice (including tomato) | 220ml of tomato juice |  |  |  |  |  |  |  |  |  |
| Other juices e.g. grape, cranberry, pineapple | 220ml of other juice |  |  |  |  |  |  |  |  |  |
| Sweetened fruit drinks e.g. cordial, squash | 220ml of cordial or squash |  |  |  |  |  |  |  |  |  |
| Sweetened water | 220ml of sweetened water |  |  |  |  |  |  |  |  |  |
| Coconut water | 220ml of coconut water |  |  |  |  |  |  |  |  |  |
| Kombucha | 220ml of plain or flavoured kombucha |  |  |  |  |  |  |  |  |  |
| Water kefir | 220ml of plain or flavoured water kefir |  |  |  |  |  |  |  |  |  |
| Sports or energy drink | 250ml or one slim can of energy drink |  |  |  |  |  |  |  |  |  |
| Dairy-based protein shake or dairy meal replacement drink | 220ml of dairy-based protein shake |  |  |  |  |  |  |  |  |  |
| Non-dairy protein shake or non-dairy meal replacement drink | 220ml of non-dairy protein shake |  |  |  |  |  |  |  |  |  |
| Other beverages e.g. grain drinks, non-alcoholic alternatives | 330ml or a standard can of other beverages |  |  |  |  |  |  |  |  |  |
| I didn't drink any of the above |  |  |  |  |  |  |  |  |  |  |

### Alcoholic beverages

| **Drink** | **Serving description** | **Average use over past month** | | | | | | | | |
| --- | --- | --- | --- | --- | --- | --- | --- | --- | --- | --- |
| **Please select which types of alcoholic beverages you've drank in the past month.**  If you didn't drink any alcoholic beverages in the past month, select 'I didn't drink any of the above' at the bottom of the page | | 1 time in the past month | 2-3 times in the past month | 1-2 times per week | 3-4 times per week | 5-6 times per week | 1 time per day | 2-3 times per day | 4-5 times per day | 6 or more times per day |
| Regular or light alcoholic beer | 330ml or one small standard can of beer |  |  |  |  |  |  |  |  |  |
| Alcoholic cider | 330ml or one small standard can of cider |  |  |  |  |  |  |  |  |  |
| Red wine | 125ml of red wine |  |  |  |  |  |  |  |  |  |
| White wine or champagne | 125ml of white wine or champagne |  |  |  |  |  |  |  |  |  |
| White spirits e.g. gin, vodka | 25ml or one shot of white spirits |  |  |  |  |  |  |  |  |  |
| Dark spirits e.g. dark rum, whiskey | 25ml or one shot of dark spirits |  |  |  |  |  |  |  |  |  |
| Other alcohol e.g. cocktails, other alcoholic drinks | 220ml of a cocktail or other alcoholic drink |  |  |  |  |  |  |  |  |  |
| Rose wine | 125ml of rose wine |  |  |  |  |  |  |  |  |  |
| I didn't drink any of the above |  |  |  |  |  |  |  |  |  |  |

## Fruit

**Fruit is up next.**

| **Food** | **Serving description** | **Average use over past month** | | | | | | | |
| --- | --- | --- | --- | --- | --- | --- | --- | --- | --- |
| **Please select which types of fruits you've eaten in the past month.**  If you didn't eat any fruit in the past month, select 'I didn't eat any of the above' at the bottom of the page | | 1 time in the past month | 2-3 times in the past month | Once a week | 2 times a week | 3-4 times a week | 5-6 times a week | Once a day | 2 or more times a day |
| Apple e.g. fresh apple, apple sauce, cooked apple | one apple, half a cup of apple sauce or cooked apple |  |  |  |  |  |  |  |  |
| Apricot | one apricot |  |  |  |  |  |  |  |  |
| Banana | one banana |  |  |  |  |  |  |  |  |
| Grapefruit | half a grapefruit |  |  |  |  |  |  |  |  |
| Grapes | a small bunch of grapes |  |  |  |  |  |  |  |  |
| Kiwi | one kiwi |  |  |  |  |  |  |  |  |
| Mango | one medium sized whole mango |  |  |  |  |  |  |  |  |
| Melon e.g. honeydew, cantaloupe, watermelon | four small pieces or one medium slice of melon |  |  |  |  |  |  |  |  |
| Nectarine | one nectarine |  |  |  |  |  |  |  |  |
| Orange, clementine, satsuma, or tangerine | half an orange, one satsuma, clementine or tangerine |  |  |  |  |  |  |  |  |
| Peach | one peach |  |  |  |  |  |  |  |  |
| Pear | one pear |  |  |  |  |  |  |  |  |
| Pineapple | seven small pieces of pineapple or four rings of canned pineapple |  |  |  |  |  |  |  |  |
| Plum | one plum |  |  |  |  |  |  |  |  |
| Blackberries | fifteen blackberries |  |  |  |  |  |  |  |  |
| Blueberries | half a cup of blueberries |  |  |  |  |  |  |  |  |
| Cherries | a half cup of cherries |  |  |  |  |  |  |  |  |
| Raspberries | fifteen raspberries |  |  |  |  |  |  |  |  |
| Strawberries | six strawberries |  |  |  |  |  |  |  |  |
| Dried fruit e.g. raisins, fruit in muesli | a small handful or one heaped tablespoon of dried fruit |  |  |  |  |  |  |  |  |
| Prunes | small handful of prunes or six stewed prunes |  |  |  |  |  |  |  |  |
| Dates | two small or one large date |  |  |  |  |  |  |  |  |
| Pomegranate | half a cup of pomegranate seeds |  |  |  |  |  |  |  |  |
| Other fruit e.g. fruit salad, mixed fruit, other fruits | half a cup of other fruit |  |  |  |  |  |  |  |  |
| I didn't eat any of the above |  |  |  |  |  |  |  |  |  |

## Vegetables, beans and legumes

**In this final section we'll ask you about vegetables, beans and legumes.**

We'll split these into:

1. Green vegetables

2. Other vegetables

3. Beans and legumes

### Green vegetables

| **Food** | **Serving description** | **Average use over past month** | | | | | | | |
| --- | --- | --- | --- | --- | --- | --- | --- | --- | --- |
| **Please select which types of green vegetables you've eaten in the past month.**  If you didn't eat any green vegetables in the past month, select 'I didn't eat any of the above' at the bottom of the page | | 1 time in the past month | 2-3 times in the past month | Once a week | 2 times a week | 3-4 times a week | 5-6 times a week | Once a day | 2 or more times a day |
| Artichoke | a third of a cup of artichokes or one globe heart |  |  |  |  |  |  |  |  |
| Asparagus | half a cup of thin stemmed asparagus, or four large asparagus spears |  |  |  |  |  |  |  |  |
| Avocado or guacamole | half an avocado or a heaped tablespoon of guacamole |  |  |  |  |  |  |  |  |
| Broccoli | one cup of broccoli |  |  |  |  |  |  |  |  |
| Brussel sprouts | six brussel sprouts |  |  |  |  |  |  |  |  |
| Celery | two to four sticks of celery |  |  |  |  |  |  |  |  |
| Cucumber | three slices of cucumber or average amount in a salad |  |  |  |  |  |  |  |  |
| Herbs e.g. coriander (UK) or cilantro (US), parsley, mint, basil | a small bunch of herbs |  |  |  |  |  |  |  |  |
| Kale | a quarter of a cup of cooked kale |  |  |  |  |  |  |  |  |
| Leeks | four pieces of leek |  |  |  |  |  |  |  |  |
| Lettuce e.g. both light and dark varieties | four leaves of lettuce |  |  |  |  |  |  |  |  |
| Peas e.g. green peas, sugar snap | three tablespoons or half a cup of peas |  |  |  |  |  |  |  |  |
| Spinach | half a cup of cooked spinach or three cups of fresh spinach |  |  |  |  |  |  |  |  |
| Green beans or runner beans | half a cup of green beans |  |  |  |  |  |  |  |  |
| Watercress, rocket or arugula | one cup of rocket |  |  |  |  |  |  |  |  |
| Courgette (UK) or zucchini (US), or marrow | five slices or a half cup of courgette (UK) or zucchini (US) |  |  |  |  |  |  |  |  |
| Other green vegetables e.g. spring greens, cavolo nero, chard | half a cup of other green vegetables |  |  |  |  |  |  |  |  |
| I don't eat any of the above |  |  |  |  |  |  |  |  |  |

### Other vegetables and vegetable dishes

**Next up are the other types of vegetables.**

| **Food** | **Serving description** | **Average use over past month** | | | | | | | |
| --- | --- | --- | --- | --- | --- | --- | --- | --- | --- |
| **Please select which types of other vegetables and vegetable dishes you've eaten in the past month.**  If you didn't eat any other vegetables or vegetable dishes in the past month, select 'I didn't eat any of the above' at the bottom of the page | | 1 time in the past month | 2-3 times in the past month | Once a week | 2 times a week | 3-4 times a week | 5-6 times a week | Once a day | 2 or more times a day |
| Beetroot | one small whole beetroot, four slices or a quarter of a cup of beetroot |  |  |  |  |  |  |  |  |
| Cabbage | half a cup of boiled cabbage |  |  |  |  |  |  |  |  |
| Carrot | 8 to 10 carrot sticks |  |  |  |  |  |  |  |  |
| Cauliflower | three large pieces or one cup of cooked cauliflower, one and a half cups of raw cauliflower |  |  |  |  |  |  |  |  |
| Coleslaw | two heaped tablespoons of coleslaw |  |  |  |  |  |  |  |  |
| Sweetcorn e.g. on the cob, tinned, frozen | 3 tablespoons or a half cup of sweetcorn, one corn fritter |  |  |  |  |  |  |  |  |
| Aubergine (UK) or eggplant (US) | five pieces or half a large aubergine (UK) or eggplant (US) |  |  |  |  |  |  |  |  |
| Garlic | one clove of garlic |  |  |  |  |  |  |  |  |
| Mushroom | two thirds of a cup of cooked mushrooms, nine small or three large raw mushrooms |  |  |  |  |  |  |  |  |
| Onion | one small onion |  |  |  |  |  |  |  |  |
| Parsnip | five pieces or half a cup of parsnip |  |  |  |  |  |  |  |  |
| Chilli pepper | one chilli pepper |  |  |  |  |  |  |  |  |
| Bell pepper or capsicum | one cup of cooked or one large raw bell pepper or capsicum |  |  |  |  |  |  |  |  |
| Radish | two radishes |  |  |  |  |  |  |  |  |
| Sprouts e.g. beansprouts, broccoli sprouts, lentil sprouts | one tablespoon of sprouts |  |  |  |  |  |  |  |  |
| Squash or pumpkin | two thirds of a cup of squash |  |  |  |  |  |  |  |  |
| Sweet potato | one medium sized sweet potato |  |  |  |  |  |  |  |  |
| Homemade or store-bought tomato sauce, tinned tomatoes or passata | half a cup of tomato sauce, tinned tomatoes or passata |  |  |  |  |  |  |  |  |
| Tomato e.g. raw, cooked, salsa | one small standard tomato |  |  |  |  |  |  |  |  |
| Turnip or swede | quarter of a cup of cooked turnip or swede |  |  |  |  |  |  |  |  |
| Gherkins, pickles or pickled vegetables | two gherkins, a tablespoon of pickled vegetables |  |  |  |  |  |  |  |  |
| Kimchi | two tablespoons of kimchi |  |  |  |  |  |  |  |  |
| Sauerkraut | two tablespoons of sauerkraut |  |  |  |  |  |  |  |  |
| Seaweed e.g. nori | one sheet of nori |  |  |  |  |  |  |  |  |
| Vegetable soup e.g. plain, mixed, with noodles | one cup of soup |  |  |  |  |  |  |  |  |
| Olives e.g. green, black | three olives |  |  |  |  |  |  |  |  |
| Other vegetable dishes e.g. vegetables with meat, fish, dairy or grains | one and a half cups of other vegetable dishes |  |  |  |  |  |  |  |  |
| Other vegetables e.g. mixed vegetables, other vegetables | half a cup of other vegetables |  |  |  |  |  |  |  |  |
| I didn't eat any of the above |  |  |  |  |  |  |  |  |  |

### Beans and legumes

**Only beans and legumes left to go.**

| **Food** | **Serving description** | **Average use over past month** | | | | | | | |
| --- | --- | --- | --- | --- | --- | --- | --- | --- | --- |
| **Please select which types of beans and legumes you've eaten in the past month.**  If you didn't eat any beans or legumes in the past month, select 'I didn't eat any of the above' at the bottom of the page | | 1 time in the past month | 2-3 times in the past month | Once a week | 2 times a week | 3-4 times a week | 5-6 times a week | Once a day | 2 or more times a day |
| White beans e.g. cannellini beans, butter beans | one heaped tablespoon of white beans |  |  |  |  |  |  |  |  |
| Black beans | one heaped tablespoon of black beans |  |  |  |  |  |  |  |  |
| Pinto beans | one heaped tablespoon of pinto beans |  |  |  |  |  |  |  |  |
| Kidney beans | one heaped tablespoon of kidney beans |  |  |  |  |  |  |  |  |
| Mung beans | one heaped tablespoon of mung beans |  |  |  |  |  |  |  |  |
| Refried beans | one heaped tablespoon of refried beans |  |  |  |  |  |  |  |  |
| Baked beans | just under one standard tin or one and a half cups of baked beans |  |  |  |  |  |  |  |  |
| Chickpeas | two to three tablespoons of chickpeas |  |  |  |  |  |  |  |  |
| Hummus | two tablespoons of hummus |  |  |  |  |  |  |  |  |
| Lentils | two to three tablespoons of lentils |  |  |  |  |  |  |  |  |
| Miso | one teaspoon of miso |  |  |  |  |  |  |  |  |
| Other beans and legumes e.g. falafel, bean dishes, bean soup | eight falafels, one cup of bean dish or soup |  |  |  |  |  |  |  |  |
| I didn't eat any of the above |  |  |  |  |  |  |  |  |  |
